# Biomechanical Mechanisms of Improved Balance Recovery to Repeated Backward Slips Simulated by Treadmill Belt Accelerations in Young and Older Adults

**DOI:** 10.3389/fspor.2021.708929

**Published:** 2021-09-21

**Authors:** Héloïse Debelle, Constantinos N. Maganaris, Thomas D. O'Brien

**Affiliations:** Research Institute for Sport and Exercise Sciences, Liverpool John Moores University, Liverpool, United Kingdom

**Keywords:** gait perturbation, balance, recovery mechanisms, age, kinetics, temporo-spatial variables, slip, step length

## Abstract

**Aim:** Exposure to repeated gait perturbations improves the balance of older adults (OAs) and decreases their risks of falling, but little is known about the underpinning mechanical adjustments. We aimed to quantify the changing temporo-spatial and kinetic characteristics of balance recovery following repeated backward slips to better understand the mechanical adjustments responsible for improved balance.

**Methods:** We exposed 17 young adults (YAs) (25.2 ± 3.7 years) and 17 OAs (62.4 ± 6.6 years) to 10 backward slips simulated on an instrumented treadmill by unilateral backward belt accelerations. We measured the balance of the participants (margin of stability: MoS), balance recovery (n_steps_: number of steps necessary to return to a steady gait for at least three consecutive steps), temporo-spatial (step length), and kinetics [ground reaction force (GRF) angle, lower limb joint moments] for 15 steps following each slip. The results were compared with baseline.

**Results:** Participants in both groups improved their MoS and n_steps_ with repeated exposure to the slips, but no significant effect of age was detected. During the perturbed step, the GRF vector was directed more posteriorly during mid-stance and more anteriorly during push-off than baseline, which resulted in a longer step. These adjustments were maintained from the first (Slip01) to the last (Slip10) slip, and by Slip10 were correlated with better balance (MoS) on the second recovery step. During the first recovery step following Slip01, participants developed lower plantarflexor and larger knee extensor moments whilst taking a shorter step, these adjustments were correlated with poorer balance and were not maintained with repeated slips. Joint moments and step length of the first recovery step returned to normal levels by Slip10.

**Conclusion:** Young adults and OAs improved their balance with repeated slips. The adjustments that were positively correlated with balance (changes in step length, GRF angle) were maintained whilst those that were not (changes in joint moments) were discarded. All the responses observed in Slip10 were observed in Slip01. The observed balance improvements were achieved by refining the initial strategy rather than by developing a new one. The underlying mechanics were correlated with step length of the first recovery steps, which was associated with balance and should be monitored in fall prevention interventions.

## Introduction

Older adults are at greater risk of falling than young adults (YAs), and these falls can result in life-threatening injuries (Spaniolas et al., 2010). Especially for community-dwelling older adults (OAs), most of the falls are triggered by trips or slips (Berg et al., 1997). Although some inconsistencies in the definitions of trips and slips exist, trips can be described as gait perturbations resulting from the sudden arrest of the swing foot that triggers a forward loss of balance, and slips as perturbations to balance resulting from sliding of the stance foot over the ground. Typically, slips arise either when the stance foot slides forward mainly shortly after heel strike (in this article: forward slips), or when the stance foot slides backward typically from mid to end stance (in this article: backward slips). Historically, backward slips have been considered less dangerous, as an individual has the opportunity to quickly regain balance with the contralateral foot. However, when participants walked on a contaminated oily surface, backward slips (*n* = 20) were observed up to 2.5 times more often than forward slips (*n* = 8) (Nagano et al., 2013). Additionally, when investigating the dangerousness of slips, Myung (2003) reported that half of the observed backward slips (5 out of 10 slips) were classified as dangerous (were arrested by a fall arresting system) when only 4 out of 14 forward slips triggered a dangerous fall. Therefore, backward slips and their recovery strategies require further attention.

Recent studies on recovery from gait perturbations show that large internal joint moments are required in response to backward slips (Debelle et al., 2020), forward slips (Yoo et al., 2019), and trips (King et al., 2019) to arrest the abnormally large angular momentum and regain control of the centre of mass (COM) position and velocity. Accordingly, the age-related deterioration in plantarflexor and knee extensor muscles' strength and tendons' reduced stiffness has been correlated with impaired balance in static (Onambele et al., 2006) and dynamic (Karamanidis et al., 2008) conditions, and linked to poorer control of the body angular momentum following trips (Pijnappels et al., 2005b). However, even though resistance training interventions successfully improve balance in static and dynamic situations (for review, see Chang et al., 2004; Papa et al., 2017), they do not necessarily directly transfer to better balance recovery when OAs are exposed to gait perturbations (Pijnappels et al., 2008). This has led to hypothesise that task-specific training (i.e., exposure to simulated slip- or trip-like perturbations) may be more beneficial than resistance exercise (for review, see Grabiner et al., 2014). The rationale for developing such interventions is that they better mimic the sensory feedback experienced during real, outside lab environment, perturbations than resistance training. They could potentially be used to adapt well-known motor schemes (here, gait pattern) to closely match the requirements induced by the change to compensate (in the present context: the perturbation), and this new behaviour could be retained and automatised for future exposure to similar conditions (Doyon and Benali, 2005). This may also be efficacious for OAs, as the ability to learn new motor skills is maintained with ageing (Durkina et al., 1995; Boyke et al., 2008; Pai et al., 2010). Further supporting the advantages of task-specific over resistance training interventions, OAs exposed to both interventions did not display better improvements than those exposed to only task-specific training (Epro et al., 2018b). Fall recovery training protocols have confirmed the ability of young and older adults to improve their balance following exposure to multiple perturbations, within one session (Konig et al., 2019b; McCrum et al., 2020) and in the long term (Bhatt et al., 2012; Epro et al., 2018b), although when compared with YAs, long-term retention appears less efficacious in OAs (Konig et al., 2019b).

To implement fall recovery training interventions, it is necessary to use protocols that apply perturbations that are as realistic as possible. Diverse protocols have been developed to achieve this, including, among others, movable low-friction platforms (Bhatt et al., 2012; Okubo et al., 2018), or split-belt instrumented (SBI) treadmills to study trips (King et al., 2019), forward slips (Yoo et al., 2019) and backward slips (McCrum et al., 2018; Debelle et al., 2020). To optimise the delivery of these protocols in fall prevention interventions, it is necessary to understand the underlying biomechanics of successful fall recovery strategies and the evolution of these strategies that result in improved balance recovery following repeated exposures. By understanding the mechanisms underlying an optimal recovery strategy, we might be able to design interventions that will specifically target these mechanisms and might be coachable outside lab environments to a wider public. To date, the mechanisms underlying balance recovery strategies with repeated perturbations have not been fully investigated, partly because of the relative novelty of this field, and also because of difficulties in recording complete kinematic, kinetic, and temporo-spatial data sets from multiple consecutive steps. In this regard, protocols utilising SBI treadmills are advantageous, because they can produce sudden unanticipated perturbation of the foot during stance, and record rich data sets during recovery.

In our previous study documenting the biomechanics of recovery from backward slips simulated by belt accelerations (Debelle et al., 2020), we detailed a protocol developed in our lab to trigger single backward slips in YAs using an SBI treadmill. We reported that in response to an induced backward slip, YAs needed four recovery steps to return their balance to normal levels, increased the length of their base of support during the perturbation by about 8% and decreased it on the following step by about 21%, and developed larger hip (+125% at peak hip extensor moment) and knee (+200% at peak knee extensor moment) moments and lower plantarflexor moments (−25% at peak plantarflexor moment) on the first recovery step, than in typical gait. As balance recovery has been shown to improve with repeated backward slip-like perturbations in YAs and OAs (McCrum et al., 2020), it is possible that the mechanical responses to a single backward slip that we previously measured might change with repeated exposures as the recovery strategy improves.

Therefore, the goal of this study was to establish whether and how young and older adults modified their gait pattern to improve their balance recovery following repeated exposure to backward slip-like perturbations. We used the aforementioned protocol to expose young and older participants to 10 repeated backward slips, and for each slip, we measured their balance on 15 recovery steps (margin of stability) and recovery of balance (n_steps_), and their kinetic and temporo-spatial variables before slip onset, during the perturbed stance and on the following recovery steps.

First, we hypothesised that both YAs and OAs would improve their balance with repeated exposures to the backward slips. Second, we hypothesised that with repeated slips the recovery strategy will be optimised to better accommodate the effects of the perturbation, through an adjustment of the recovery steps' length and a redistribution of the joint moments to rely more on the hip and knee joints. Finally, we hypothesised that OAs would develop a similar recovery strategy and recovery strategy adjustments as YA, whilst possibly needing more steps to recover their balance.

## Methods

### Participants and Protocol

Seventeen young (eight males, nine females, age 25.2 ± 3.7 years, height 176.1 ± 8.1 cm, body mass 71.8 ± 10.1 kg) and 17 older (3 males, 14 females, age 62.4 ± 6.6 years, height 161.8 ± 7.2 cm, body mass 66.5 ± 11.3 kg) adults volunteered to take part in this study. All participants were able to walk unassisted for at least 15 min, and were free from any lower limb injury in the last 6 months, surgery in the last 2 years, and balance, neurological or musculoskeletal disorders.

**Figure 1:**
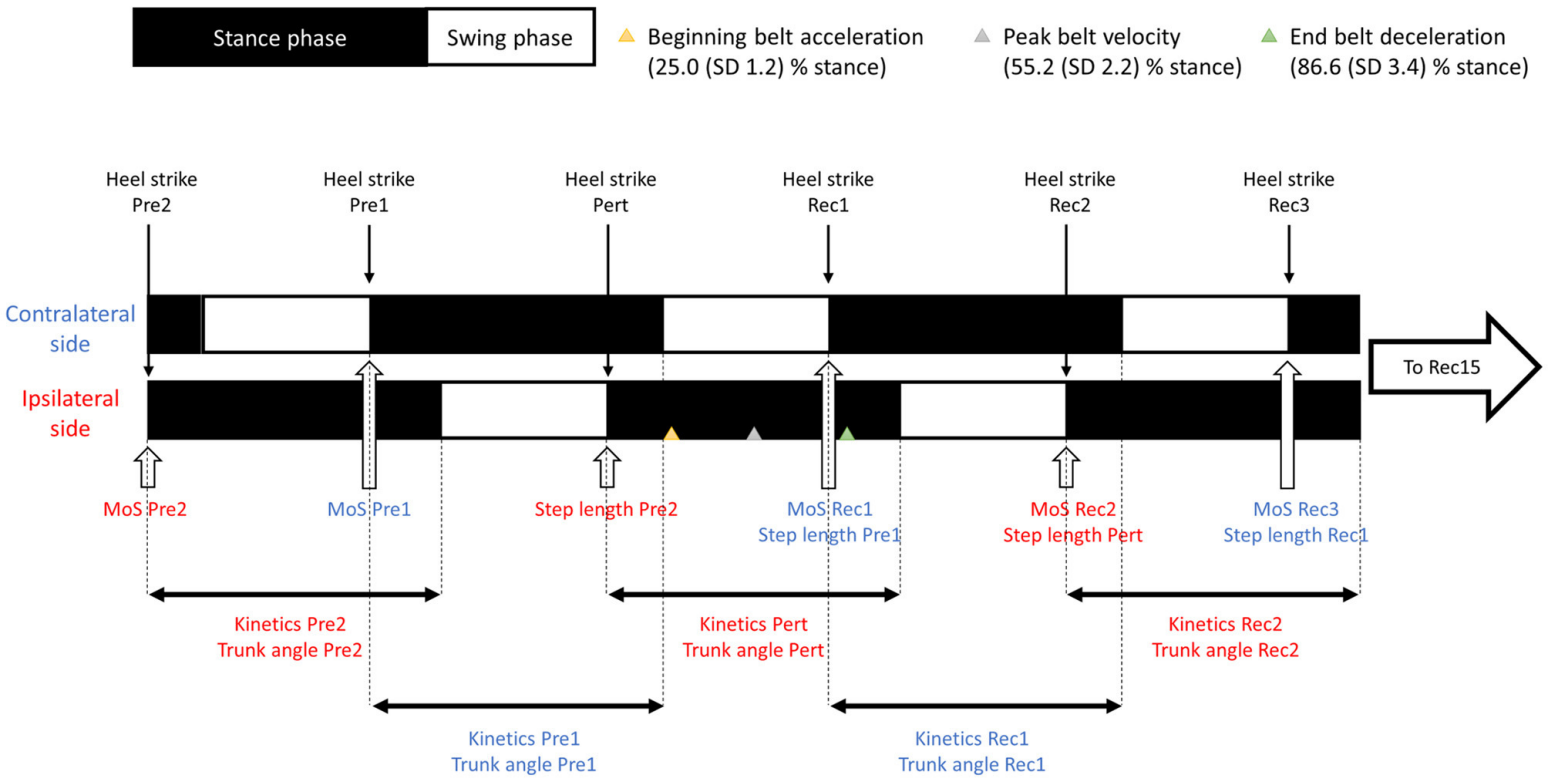
Diagram of steps and outcome measures. In red: outcome measures from the ipsilateral side to the perturbation; in blue, outcome measures from the contralateral side.

Participants were exposed to 10 backward slip-like perturbations while walking on an SBI-treadmill (300 Hz, M-Gait, Motek; Motekforce Link, Amsterdam, The Netherlands), and the protocol of the perturbation has been described in detail previously (Debelle et al., 2020). Briefly, after 5 min of familiarisation with participants walking at 1.2 m·s^−1^, we first recorded baseline data (Normal), and then triggered the perturbation at random and unexpected times by an acceleration (5 m·s^−2^) of the belt, followed by a return to normal speed. Perturbations were randomly assigned either to the right or the left side using MinimPy (http://sourceforge.net/projects/minimpy) and applied consistently to that limb. Belt accelerations were designed to start at 20% stance phase with the belt speed returning to 1.2 m·s^−1^ at 70% stance. Mechanical latency and quicker stance phase during the perturbation than in normal gait meant that the perturbation actually occurred slightly later than these timings (Figure 1). Previous results on the experimental validity of the protocol indicated a very good consistency between the timings of acceleration beginning [25 (SD 1.2) % of stance, CV = 5%] and return to 1.2 m·s^−1^ [86.5 (SD 3.4) % of stance, CV = 4%, respectively]. These accelerations produced a forward loss of balance during the second half of stance, from which the participants had to adjust to avoid falling.

For safety, participants wore a full-body safety harness attached to a frame above the treadmill. They were instructed beforehand that should they experience a trip or a slip, they should try to recover their balance and continue walking as if they had experienced one outside of the lab. The participants were also asked to avoid using the handrails, and although vigorous arm movements were occasionally observed, none grabbed the handrails. To ensure that participants' balance had returned to normal levels, participants continued walking on the treadmill for 1 to 2 min before the next perturbation trigger. This was repeated until 10 perturbations had been triggered.

Data were recorded for both the ipsilateral (Pre2) and contralateral (Pre1) steps prior to the perturbed step (Pert) and up to the 15th recovery step (Rec15). Zero-dimensional data (margin of stability and step length) were measured at heel strike and one-dimensional data (kinetics and temporo-spatial) over 100% of stance phase (Figure 1).

Since the primary aim of this study was to determine the mechanisms by which the participants achieved better recovery and a very large data set was developed during the experiment, it was necessary to include in the main manuscript only the results that helped achieving this aim. Therefore, we only report kinetics and temporo-spatial results (1) if they were significantly different from normal and (2) if they were correlated to balance recovery. Results not meeting these criteria are reported in the Supplementary Material.

This study was carried out with the approval of the Liverpool John Moores University and National Health Service (NHS) ethics committees (18/NW/0700). Written consent was obtained in accordance with the declaration of Helsinki.

### Evaluation of Balance

Participants' balance was quantified from the margin of stability (MoS) (Hof et al., 2005), measured as the distance between the anterior boundary of the base of support (BoS) (anterior-posterior position of the second toe marker of the leading foot) and the extrapolated centre of mass (XCoM) at heel strike. A positive MoS indicated that the XCoM was located behind the anterior boundary of the BoS and that the participant was stable. Balance was assessed in the two steps prior to each slip (Pre2 and Pre1) to test for changes in walking pattern with repeated slips resulting from the anticipation of a potential upcoming perturbation due to any sensory cue (visual, auditory, or vibration). Balance was also assessed for 15 recovery steps following the slip (Rec1-15) to establish the time course of balance recovery. The MoS data are reported as mean ± SD. For Normal condition, MoS SD was computed as the average of each participant's SD on that trial, for the other trials, MoS SD was computed as the group's SD.

The position and velocity of the feet's markers and participants' COM were computed and filtered using a low-pass fourth-order Butterworth filter with a cut-off frequency of 8 Hz in Visual3D (C-Motion; Germantown, MD, United States) before being exported to Matlab (R2020; MathWorks, Natick, MA, United States) for calculation of the MoS.

To quantify how long it took the participants to recover their balance following each slip, we quantified n_steps_ as the first step of at least three consecutive steps within one standard deviation of normal MoS, which was determined as the average of five gait cycles recorded during steady gait on the treadmill after the familiarisation period. When participants did not reach stable gait by the last recorded step (Rec15), we set n_steps_ to 16 (n_steps_ was set at 16 for 14 participants (7 YAs and 7 OAs) during Slip01, and for 4 participants (2 YAs and 2 OAs) during Slip10).

### Mechanics of Recovery

We used a 6DoF marker set with 68 retroreflective markers tracked by 12 motion capture cameras (120 Hz; Vicon Motion Systems, Oxford, United Kingdom) to measure three-dimensional whole-body kinetic and kinematic data while the participants were walking on the treadmill. Force data were recorded using Vicon at a sample rate of 1,200 Hz.

We evaluated changes in trunk angle (sagittal plane) and step length (anterior-posterior distance between the centres of mass of each foot at heel strike of the leading foot) for each step of each slip trial. These parameters were chosen, as they could be easily targeted in a fall prevention intervention. To allow comparisons between the participants, step length was computed in percentage of body height (% BH). To understand how participants adapted their gait pattern between the first and last slips, we measured the internal joint moments at the hips, knees, and the ground reaction force angle to the vertical (GRF_θ_, + = anterior) as the inverse tangent of the ratio between the anterior-posterior and vertical GRF vectors.

Kinetics (joint moments) and kinematics (trunk angle) data were computed in Visual3D, using inverse dynamics for the joint moments, and filtered using a low-pass fourth-order Butterworth filter with a cut-off frequency of 8 Hz. The same filter was used on the anterior-posterior and vertical GRF vectors and temporo-spatial (location of the feet's COM) data that were then exported to Matlab where the GRF_θ_ and step length were computed.

### Statistical Analysis

All the variables were tested for normality by Shapiro–Wilk's test.

To test whether the participants changed their gait pattern in anticipation of the slip, we compared the MoS, kinetic and temporo-spatial variables during normal with Pre2 and Pre1 of Slip01 and Slip10. When main effects of Age (YAs, OAs) or Conditions (Normal, Slip01_Pre2, Slip01_Pre1, Slip10_Pre2, Slip10_Pre1) were detected, Bonferroni *post hoc* tests were performed and alpha adjusted to the number of tests (α = 0.01 or α = 0.0063, respectively). For the MoS, step length, and joint moments during Pre, we performed non-parametric tests [Mann2Whitney (Age: YAs, OAs), Friedman (Conditions: Slip01 to Slip10), and Wilcoxon signed rank tests for *post-hoc* comparisons], and parametric mixed-design ANOVAs for the trunk angle.

To test whether participants' balance was different from normal following each slip, we compared the MoS of each recovery step (Rec1–Rec15) with Normal using mixed-design ANOVAs: Age (YA, OA), Conditions (for each slip trial: Normal, Rec1 to Rec15), Age^*^Conditions. Because we repeated the analysis 10 times, α was adjusted to 0.005.

To test for differences in the number of recovery steps required to return to normal balance with repeated slips, we compared n_steps_ between each slip trial using Mann-Whitney (Age: YAs, OAs), Friedman (Conditions: Slip01 to Slip10), and Wilcoxon signed rank tests for *post-hoc* comparisons with Bonferroni adjustments (α = 0.0011).

To test for differences in the biomechanics of recovery following the first (Slip01) and last (Slip10) slips, we evaluated the changes in the reactive kinetic and temporo-spatial variables measured during the perturbation and the first (Rec1) and second (Rec2) recovery steps of Slip01 and Slip10. The following conditions were included in the analysis: Normal, Slip01_Pert (perturbed step of Slip01), Slip01_Rec1 (first recovery step of Slip01), Slip01_Rec2 (second recovery step of Slip01), Slip10_Pert, Slip10_Rec1, and Slip10_Rec2. Although the perturbed step cannot be considered as a recovery step per se, we included it in the present analysis to then evaluate whether and how changes in the biomechanics during the perturbation affected the balance and balance recovery in the following steps. When a main effect of Age was detected, Bonferroni *post-hoc* tests were performed and alpha adjusted to the number of tests (α = 0.007). When a main effect of Condition was detected, Bonferroni *post-hoc* tests were performed (n =9: Normal vs. Pert, Rec1, and Rec2 for both Slip01 and Slip10, Slip01 vs. Slip10 in Pert, Rec1, and Rec2) and alpha adjusted to the number of tests performed (α = 0.0056).

Finally, when we found a significant effect of condition on the MoS in the Pre steps, kinetics, kinematics and temporo-spatial variables, we used bivariate parametric and non-parametric correlations to understand whether and how these variables affected the balance (MoS) on the following recovery steps, and the balance recovery (n_steps_) on that trial. Specifically, we ran a correlation analysis to understand (1) whether and how participants' balance (MoS) prior to the slip was related to participants' balance following the slip, (2) whether and how participants' balance (MoS or n_steps_) in the first slip trial was related to the balance in the last slip trial, and (3) whether and how the kinetic, kinematic, and temporo-spatial adjustments made when recovering from Slip01 and Slip10 affected the MoS of the next recovery steps and n_steps_ on that slip. For (1), we ran a correlation analysis between the MoS during Slip01_Pre2 and Slip01_Rec1, Slip01_Pre1 and Slip01_Rec1, Slip10_Pre2 and Slip10_Rec1, and between Slip10_Pre1 and Slip10_Rec1. For (2), we ran a correlation analysis to understand whether the MoS of Slip01_Rec1 and Slip01_Rec2 was correlated with the MoS of Slip10_Rec1 and Slip10_Rec2, respectively, and whether n_steps_ of Slip01 was correlated with n_steps_ of Slip10. For (3), when a kinetic, kinematic, or spatio-temporal variable was significantly different from Normal levels, we ran a correlation analysis to evaluate whether this variable was related to the MoS of the next steps or to n_steps_ of that slip trial. To use one-dimensional variables (kinetic and temporo-spatial variables for which a significant effect of condition was found between a step and Normal) in the correlation analysis, we used the average from the region of interest (region of significant difference from Normal as determined by statistical parametric mapping, SPM). Because n_steps_ was not normally distributed, the correlations between kinetics or temporo-spatial parameters and n_steps_ should be treated with caution. Participants whose n_steps_ was set to 16 were excluded from the correlation analysis including n_steps_.

Statistical analysis was performed using SPSS 26 (IBM, NY) for zero-dimensional data (i.e., MoS, n_steps_, and step length), and we performed statistical parametric mapping in Matlab for one-dimensional data (i.e., GRF_θ_, joint moments and trunk angle).

## Results

### Anticipatory Adjustments

**Figure 2:**
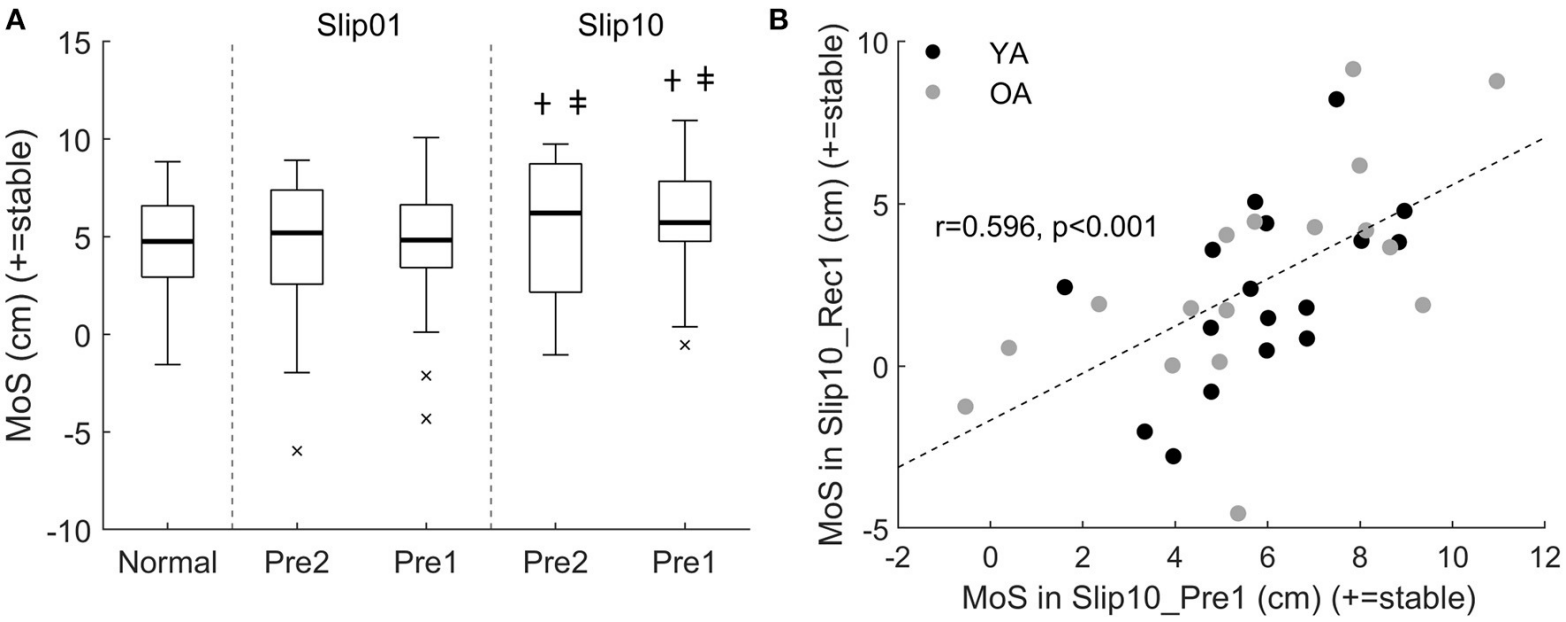
**(A)** Boxplots of the margin of stability (MoS) for all participants in the two steps prior to Slip01 and Slip10 (Pre2 and Pre1). Thick horizontal black lines: median; thin horizontal black line: first and third quartiles; × : outliers. †: Slip10 significantly higher than normal (*p* ≤ 0.005); ‡: Slip10 significantly higher than Slip01 (*p* ≤ 0.023). See Supplementary Figure 1 for participants' data points. **(B)** Correlation between MoS in Slip10_Pre1 and MoS in Slip10_Rec1, *r* = 0.596, *p* < 0.001. Black circles: young adults (YAs); grey circles: older adults (OAs).

The margin of stability at heel strike of the two steps prior to the first and last slips (Pre2 and Pre1) was not significantly different between the age groups (*p* = 0.309). Irrespective of age, there was no difference in the MoS between the first slip (Pre2 or Pre1) and Normal, but the MoS was larger in the Pre2 and Pre1 of the last slip than both Normal and the equivalent steps of the first slip (*p* ≤ 0.023, Figure 2A). These anticipatory adjustments of the MoS before the last slip were correlated with better balance following that last slip (Spearman's rho = 0.565, *p* = 0.001 between the MoS in Slip10_Pre2 and Slip10_Rec1, and *r* = 0.596, *p* < 0.001 between the MoS in Slip10_Pre1 and Slip10_Rec1, Figure 2B). No significant difference existed between Slip01_Pre2 and Slip01_Pre1 (*p* = 0.614), or between Slip10_Pre2 and Slip10_Pre1 (*p* = 0.567), suggesting that although the participants might have adjusted their balance in anticipation of a potentially upcoming perturbation, they did not anticipate its timing.

We found no main effect of either Age or Conditions on the knee and ankle moments or trunk angle during the steps preceding the perturbations (*p* > 0.05). Significant main effects of Age and Conditions existed for both step length and hip moments during the pre-slip steps, but these changes were not correlated with balance (neither the MoS of the first and second recovery steps of Slip10 nor n_steps_ in Slip10, *p* > 0.05).

### Recovery Adjustments

**Figure 3:**
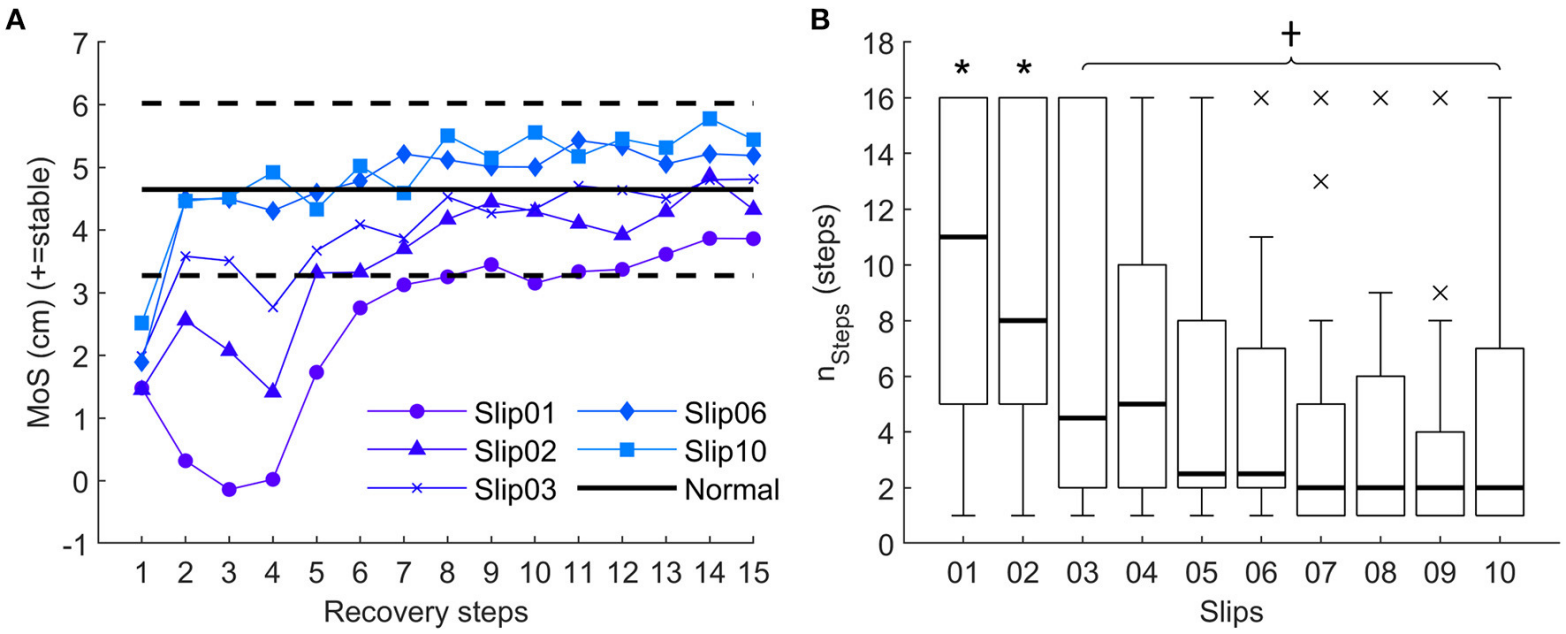
**(A)** Average margin of stability for all participants for the 15 recovery steps recorded for Slip01, Slip02, Slip03, Slip06, and Slip10. Solid and dotted horizontal black lines represent Normal ± 1 standard deviation (SD), respectively. See Supplementary Figure 2 for YA and OA curves. **(B)** Boxplots of n_steps_ (i.e. first step of at least three consecutive steps back to ± 1 SD of Normal MoS) from Slip01 and Slip10. Thick horizontal black lines: median; thin horizontal black line: first and third quartiles; × : outliers. *: significantly larger than Slip10, *p* ≤ 0.0011; †: significantly lower than Slip01, *p* ≤ 0.0011. See Supplementary Figure 3 for participants' data points.

We found no significant effect of Age on the MoS or n_steps_ (*p* > 0.005 for MoS and *p* = 0.052 for n_steps_). We found a significant effect of Conditions on the MoS from Slip01 to Slip06 (*p* < 0.005) and on n_steps_ (*p* < 0.001). A *post hoc* analysis showed that following Slip01 and Slip02, the MoS was significantly lower than Normal (MoS Normal = 4.6 ± 1.3 cm) until the sixth and fifth recovery steps, respectively (MoS Slip01_Rec6 = 2.8 ± 3.6 cm, *p* = 0.005, and MoS Slip02_Rec5 = 3.3 ± 3.6 cm, *p* < 0.001). From Slip03 to Slip06, only Rec1 had a significantly lower MoS compared with Normal (MoS Slip03_Rec1 = 2.0 ± 3.7 cm, *p* = 0.007; MoS Slip06_Rec1 = 1.9 ± 3.1, *p* = 0.001) (Figure 3A). Significant positive correlations between the MoS of Slip01_Rec1 and the MoS of Slip10_Rec1 (*r* = 0.698, *p* < 0.001), and between the MoS of Slip01_Rec2 and the MoS of Slip10_Rec2 (Spearman's rho = 0.661, *p* < 0.001) indicate that the participants who recovered well during the first slip trial tended to also recover well during the last trial.

Accordingly, n_steps_ decreased with the number of slips (n_steps_ Slip01 = 10.3 ± 5.6 steps, n_steps_ Slip10 = 4.9 ± 5 steps, *p* < 0.001) until Slip03, from which n_steps_ was not significantly larger than in Slip10 (n_steps_ Slip02 = 9.1 ± 5.5 steps, *p* < 0.001; n_steps_ Slip03 =7.2 ± 6.2 steps, *p* = 0.016; n_steps_ Slip04 = 6.1 ± 5.1 steps, *p* = 0.066). From Slip03 to Slip10, n_steps_ was constantly lower than n_steps_ in Slip01 (*p* ≤ 0.001) (Figure 3B).

Trunk angle data have not met the criteria for being reported in the main manuscript and are reported in the Supplementary Material.

**Figure 4:**
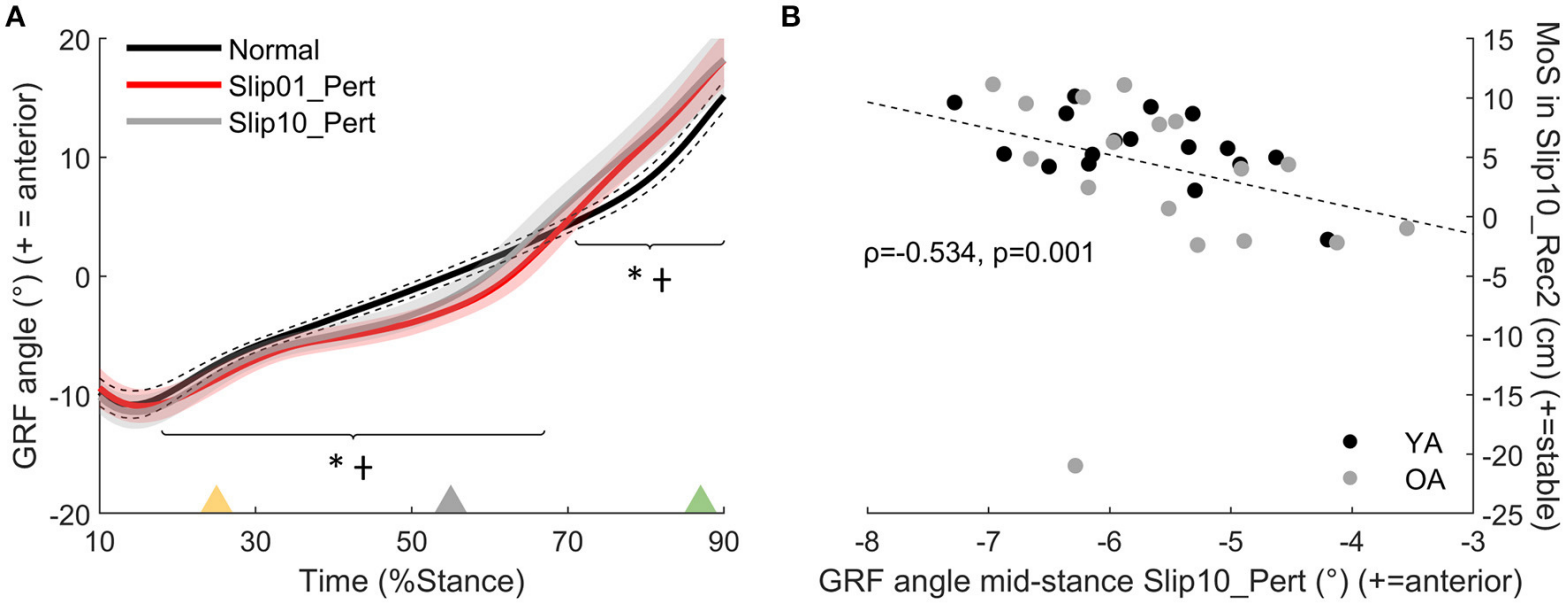
**(A)** Ground reaction force angle (GRF_θ_) (°), effect of condition between Normal (black line), Slip01_Pert (red line), and Slip10_Pert (grey line); *: Slip01 significantly different from Normal *p* < 0.001 from 18 to 67% and from 71 to 90% of stance; †: Slip10 significantly different from Normal *p* < 0.001 from 15 to 60% and from 68 to 90% of stance. Yellow triangle: beginning of belt acceleration; grey triangle: peak belt speed; green triangle: belt speed returns to 1.2 m·s^−1^. See Supplementary Figure 4 for YA and OA curves. **(B)** Correlation between GRF_θ_ during mid stance of Slip10_Pert and MoS in Slip10_Rec2 (Spearman's rho = −0.534, *p* = 0.001). Black circles: young adults; grey circles: older adults.

*During the perturbation*, the participants' sagittal GRF_θ_ was directed more posteriorly during mid stance than in Normal condition for Slip01 (*p* < 0.001 from 18 to 67% of stance, Figure 4A), and then directed more anteriorly than in Normal at the end of stance (*p* < 0.001 from 71 to 90% of stance, Figure 4A). These modifications were maintained during Slip10 (*p* < 0.001 from 15 to 60% and from 68 to 90% of stance, Figure 4A), and we found that in Slip10, the participants whose GRF_θ_ was directed more posteriorly during mid stance (averaged from 15 to 60% stance) were those who better recovered their balance during Rec2 of Slip10 (Spearman's rho = −0.534, *p* = 0.001, Figure 4B).

**Figure 5:**
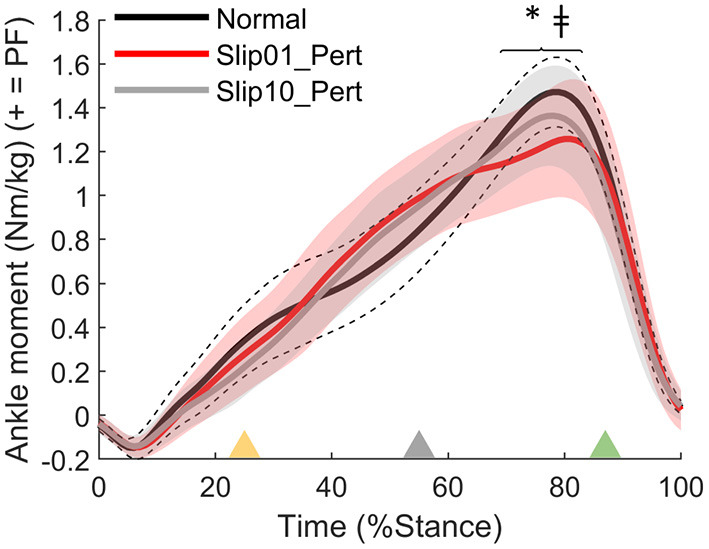
Ankle moment, effect of condition between Normal (average: solid black line; standard deviation: dotted black lines), Slip01_Pert (average: solid red line; standard deviation: red shaded area), and Slip10_Pert (average: solid grey line; standard deviation: grey shaded area). *: significant difference between Normal and Slip01 (69–83% stance, *p* = 0.002); ‡: significant difference between Slip01 and Slip10 (70–79% stance, *p* < 0.001). Yellow triangle: beginning of belt acceleration; grey triangle: peak belt speed; green triangle: belt speed returns to 1.2 m·s^−1^. See Supplementary Figure 5 for YA and OA curves.

We found that participants developed a lower than Normal plantarflexor moment during push-off of the first slip (Slip01_Pert: *p* = 0.002 from 69 to 83% stance, Figure 5), which returned to Normal levels by the last slip (*p* > 0.0056 between Slip10 and Normal; *p* < 0.001 from 70 to 79% stance between Slip01 and Slip10, Figure 5).

**Figure 6:**
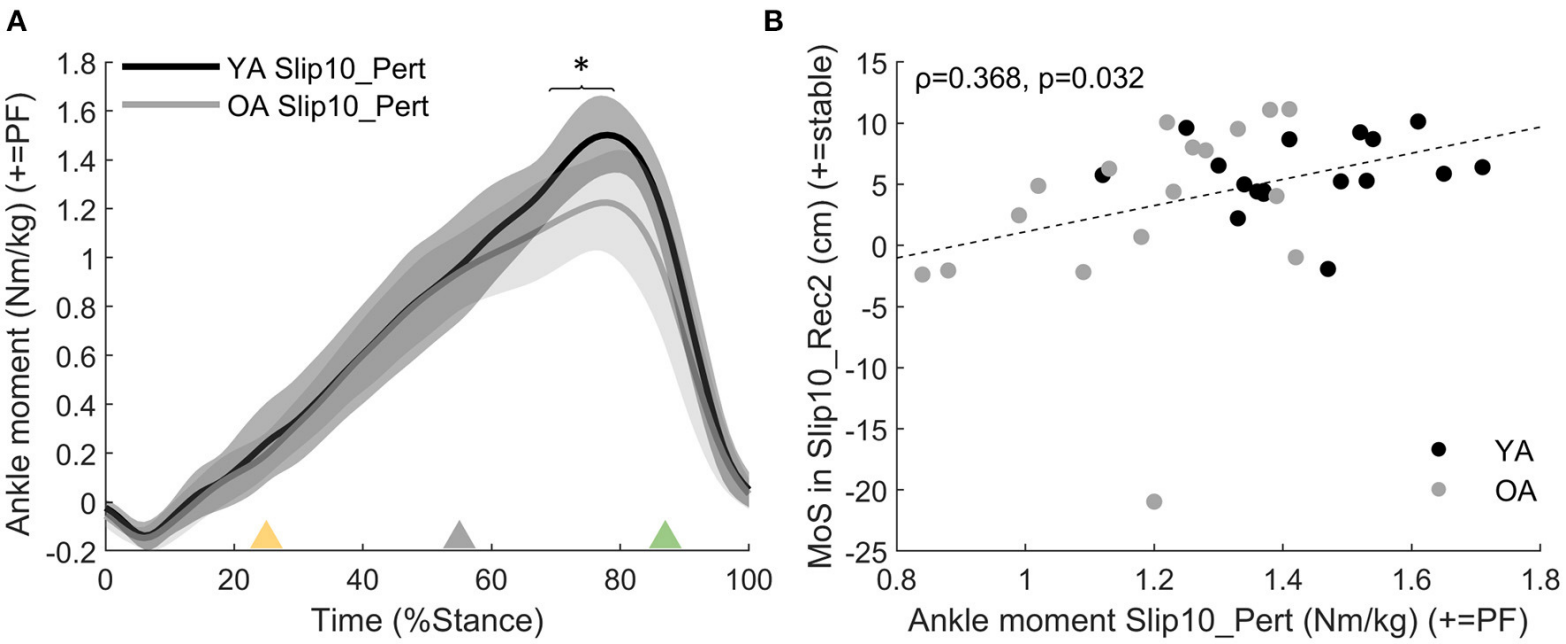
**(A)** Ankle moment age difference between YAs and OAs for Slip10_Pert. Solid black line: YA in Slip10_Pert; solid grey line: OA in Slip10_Pert. *: significant effect of Age (*p* = 0.004 from 69 to 79% stance). Yellow triangle: beginning of belt acceleration; grey triangle: peak belt speed; green triangle: belt speed returns to 1.2 m·s^−1^. **(B)** Correlation between ankle moment in Slip10_Pert (69–79% stance) and the MoS of Slip10_Rec2 (Spearman's rho = 0.368, *p* = 0.032). Black circles: young adults; grey circles: older adults.

The OAs developed a lower plantarflexor moment than YAs at push-off of the perturbed step during the last slip than YA (*p* = 0.004 from 69 to 79% stance for Slip10_Pert, Figure 6A, not significant for Slip01). During that same step, participants who developed larger plantarflexor moments were the ones with the higher MoS at heel strike of the second recovery step (Slip10_Rec2, Spearman's rho = 0.368, *p* = 0.032, Figure 6B). These suggest that the OAs, who were grouped lower on the Ankle Moment – MoS correlation graphs (Figure 6B), might be at higher risk of falling than the YAs, at least partly due to an inability to produce enough propulsive force at push-off of the leg that slipped.

**Figure 7:**
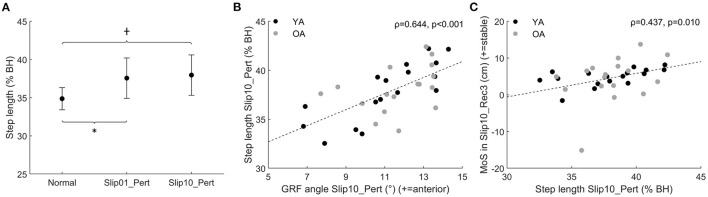
**(A)** Step length of Slip01_Pert and Slip10_Pert compared with Normal (average ± SD as error bars); *: significant difference between Slip01 and Normal, *p* < 0.001. †: significant difference between Slip10 and Normal, *p* < 0.001. See Supplementary Figure 6 for participants' data points. **(B)** Correlation between GRF_θ_ during push-off of Slip10_Pert (68–90% of stance) and step length in Slip10_Pert (Spearman's rho = 0.644, *p* < 0.001). **(C)** Correlation between step length in Slip10_Pert and MoS in Slip10_Rec3 (Spearman's rho = 0.437, *p* = 0.01). Black circles: young adults; grey circles: older adults. % BH: % body height.

On average, participants took a longer step in the first and last slips than in Normal (Slip01_Pert and Slip10_Pert, *p* < 0.001, Figure 7A). The participants who took a longer step in Slip10_Pert were those who had the GRF_θ_ directed more anteriorly in Slip10_Pert (Spearman's rho = 0.644, *p* < 0.001, Figure 7B), and those who better recovered their balance by the third recovery step of the last slip (Slip10_Rec3, Spearman's rho = 0.437, *p* = 0.01, Figure 7C).

**Figure 8:**
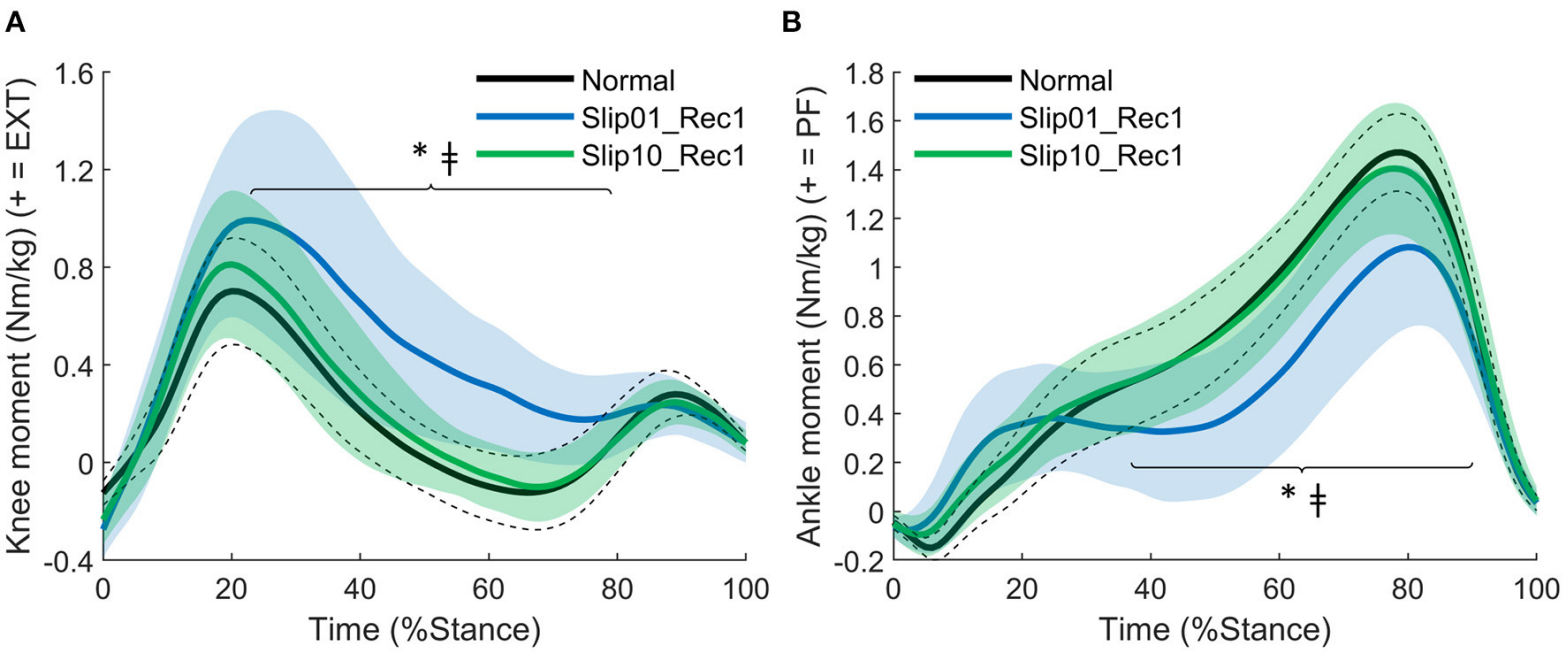
**(A)** Knee moment: effect of condition between Normal, Slip01_Rec1, and Slip10_Rec1. *: significant difference between Normal and Slip01 (23–79% stance *p* = 0.002); ‡: significant difference between Slip01 and Slip10 (32–80% stance, *p* = 0.002). **(B)** Ankle moment: effect of condition between Normal, Slip01_Rec1 and Slip10_Rec1. *, significant difference between Normal and Slip01 (37–90% stance, *p* = 0.002); ‡: significant difference between Slip01 and Slip10 (34–89% stance, *p* < 0.001). Normal: average: solid black line; SD: dotted black lines; Slip01_Rec1: average: solid blue line; SD: blue shaded area; Slip10_Rec1: average: solid green line; SD: green shaded area. See Supplementary Figure 7 for YA and OA curves.

*During the first recovery step*, participants developed a larger knee extensor moment (*p* = 0.002 from 23 to 79% stance, Figure 8A) and a lower ankle plantarflexor moment (*p* = 0.002 from 37 to 90% stance, Figure 8B) in the first slip (Slip01_Rec1) compared with Normal. These joint moments had returned to Normal levels by the last slip (*p* > 0.0056 between Slip10_Rec1 and Normal, *p* = 0.002 for knee moment from 32 to 80% of stance between Slip01_Rec1 and Slip10_Rec1 (Figure 8A), and *p* < 0.001 for ankle moment from 34 to 89% of stance between Slip01_Rec1 and Slip10_Rec1, Figure 8B).

**Figure 9:**
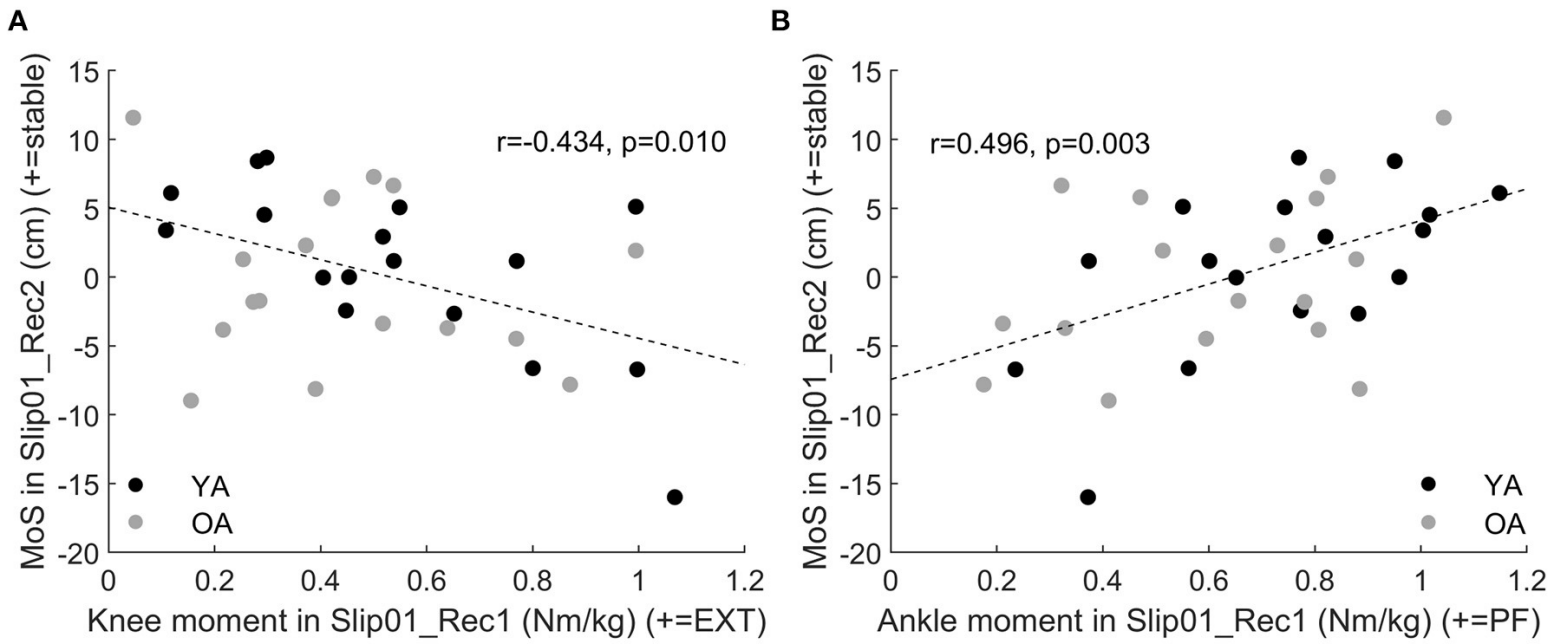
**(A)** Correlation between knee moment in Slip01_Rec1 from 23 to 79% stance and the MoS of Slip01_Rec2 (*r* = −0.434, *p* = 0.01). **(B)** Correlation between ankle moment in Slip01_Rec1 from 37 to 90% stance and the MoS of Slip01_Rec2 (*r* = 0.496, *p* = 0.003). Black circles: young adults; grey circles: older adults.

Knee and ankle extensor moments in Slip01_Rec1 had moderate to good correlations with MoS of the second recovery step (Slip01_Rec2, r = −0.434, *p* = 0.01, Figure 9A; *r* = 0.496, *p* = 0.003, Figure 9B, respectively). Therefore, participants who developed a larger knee extensor moment and a lower ankle plantarflexor moment in mid stance of Slip01_Rec1 seemed to have a poor balance during the following steps.

**Figure 10:**
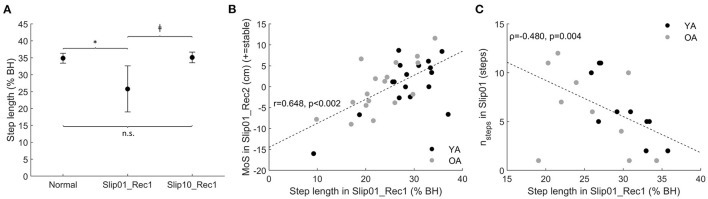
**(A)** Step length of Slip01_Rec1 and Slip10_Rec1 compared with Normal (average ± SD as error bars); *: significantly shorter than Normal, *p* < 0.001; ‡: Slip10 significantly longer than Slip01, *p* < 0.001; n.s.: no significant difference between conditions. See Supplementary Figure 8 for participants' data points. **(B)** Correlation between step length in Slip01_Rec1 and MoS of Slip01_Rec2 (*r* = 0.648, *p* < 0.001). Black circles: young adults; grey circles: older adults. **(C)** Correlation between step length in Slip01_Rec1 and n_steps_ (first of at least three consecutive steps within one standard deviation of normal MoS) in Slip01 (Spearman's rho = −0.480, *p* = 0.004), participants whose n_steps_ had not returned to Normal by Rec15 were removed from the correlation analysis. Black circles: young adults; grey circles: older adults. % BH: % of body height.

On average, participants took a smaller step in Slip01_Rec1 that returned to Normal length by the last slip (*p* < 0.001 between Slip10_Rec1 and Slip01_Rec1, Figure 10A). There were moderate to good correlations between the length of the first recovery step of the first slip (Slip01_Rec1) and the MoS of the next step (Slip01_Rec2, *r* = 0.648, *p* < 0.001, Figure 10B) and between step length in Slip01_Rec1 and n_steps_ in Slip01 (Spearman's rho = −0.48, *p* = 0.004, Figure 10C), suggesting that during the first slip participants who took a longer step in Rec1 seemed to be those who had a better balance on the following step and required fewer steps to return to a stable balance during that trial.

**Figure 11:**
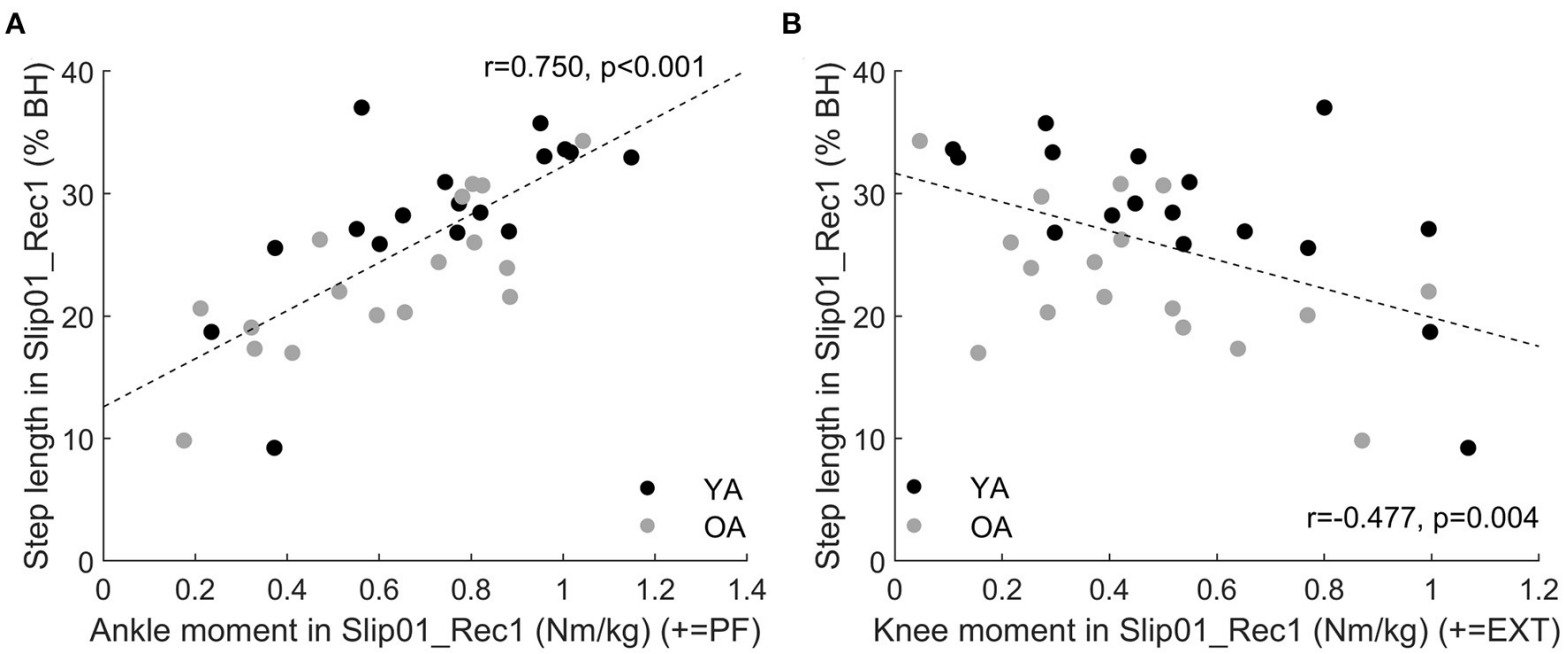
**(A)** Correlation between ankle moment in Slip01_Rec1 from 37 to 90% stance and step length in Slip01_Rec1 (*r* = 0.75, *p* < 0.001). **(B)** Correlation between knee moment in Slip01_Rec1 from 23 to 79% stance and step length in Slip01_Rec1 (*r* = −0.477, *p* = 0.004). Black circles: young adults; grey circles: older adults. % BH: % body height.

We found that the length of the first recovery step during the first slip trial (Slip01_Rec1) was correlated with ankle and knee moments during that step, with the participants who developed the larger ankle plantarflexor moment during push-off being those who took the longer step (*r* = 0.75, *p* < 0.001, Figure 11A), and those who developed the larger knee extensor moment in mid stance being the ones who took the shorter step (*r* = −0.477, *p* = 0.004, Figure 11B).

None of the variables that differed from Normal *during the second recovery step* were correlated with balance. Therefore, results related to the mechanisms of recovery in the second recovery step are reported in the Supplementary Material.

## Discussion

With this study, we showed that: (a) balance recovery following repeated slip-like perturbations simulated by treadmill belt accelerations can be improved with repeated exposure in young and older adults, which supports our first hypothesis, and more importantly, (b) the older adults demonstrated improvements that were not different to those of younger adults. Following the first slip, participants utilised biomechanical responses that were associated with both better and worse recovery. However, the recovery strategy was optimised with repeated exposures to preferentially retain only the responses associated with better recovery or which resulted in a rapid return to normal balance following the slip. Generally, this optimal recovery strategy requires changes in the orientation of the GRF vector (Figures 4A,B), length of the perturbed and recovery steps (Figures 7A, 10A), and internal moments around the knee and ankle joints (Figures 5, 8A,B). This improvement in balance recovery after repeated exposure was, in part, achieved by adopting a length for the first recovery step closer to normal, which offers an easily explained and monitored strategy to teach in fall prevention interventions. Together, these findings give further evidence that fall prevention interventions that use repeated backward slip-like perturbations on an instrumented treadmill as a form of training have the potential to be effective for this mechanism of falling.

During Slip01, the direction of the GRF vector was adjusted and the step length increased during the perturbed step; these characteristics were associated with a more optimal strategy. However, participants also developed low ankle plantarflexor moments during the slip and first recovery step, high knee extensor moments during Rec1, and took a small step during Rec1, which were all associated with poor balance recovery. The recovery strategy did not differ between the age groups; therefore, we accept the hypothesis that OAs developed a similar recovery strategy as YAs on the first slip. However, independent of age, this gross, generalised reaction to the first slip was then fine-tuned to retain only the beneficial characteristics by the 10th slip, in which the participants demonstrated a more optimal recovery strategy. Specifically, by Slip10, participants' GRF vector was still directed more posteriorly during mid stance and anteriorly during push-off, they took a longer step during the slip, generated larger plantarflexor moments compared with Slip01 during both Pert and Rec1, and had returned their knee moments and step length of Rec1 back to normal levels. We, therefore, accept the hypothesis that step length is altered with repeated backward slip-like perturbations, but reject the hypothesis that the joint moments would be redistributed to rely more on the hip and knee joints and less on the ankle joint.

This improved balance and shift towards an optimal strategy by Slip10 for both YAs and OAs validate our hypothesis on the recovery strategy developed by young and older adults, and show that task-specific perturbation training by exposure to multiple mechanical perturbations can be used as an intervention to improve balance recovery from backward slip-like perturbations, as already demonstrated elsewhere (McCrum et al., 2020). With this study, we have established biomechanical strategies by which the improvement in recovery is achieved. However, whether this can be used to reduce the risk of falling in outside-lab, real-world conditions remains to be examined for this particular type of perturbation. Particularly, we showed that keeping the step length close to normal levels was an important component of balance recovery. More studies are needed to understand whether interventions training older adults to maintain a normal step length in response to external perturbations can prevent falls in real-world conditions. However, as the step length is (1) easy to monitor outside lab settings and (2) easily understandable by participants, fall prevention interventions targeting the step length and not requiring specialised treadmills should be developed, and if successful in decreasing fall risks, could be used to reach larger cohorts.

Increased step length, as it can compensate for larger COM displacement, independently of the direction and type of perturbation, has often been associated with better balance for postural perturbations (Owings et al., 2001), trips (Okubo et al., 2018), forward slips (Patel and Bhatt, 2015), and now backward slips. The contribution of the joint moments to balance recovery, however, is task specific, as the mechanical requirements vary widely, but the general consensus tends towards the development of large internal moments at the lower limb joints as a reaction to the perturbation (Pijnappels et al., 2005a; Liu and Lockhart, 2009; King et al., 2019; Yoo et al., 2019). Surprisingly, in this study, we found that optimal recovery strategy did not require the development of larger than normal joint moments, and that large knee extensor moments were actually correlated with poor balance. In the aforementioned studies, the adjustments of the joint moments in response to repeated perturbations were not accounted for, neither were the joint moments directly correlated with balance performance. Therefore, the effect of these kinetic changes on fall avoidance and balance recovery was assumed but not actually demonstrated. We cannot rule out that large joint moments may be important for recovery from perturbations more mechanically demanding than the one we applied, but as reported in the Supplementary Material, hip moments during the perturbed stance and the first recovery step, as well as knee moments during the perturbed step, were larger than in normal condition and did not return to normal levels by Slip10. As we could not link these changes to improved balance recovery, we reject the hypothesis that the kinetics of improved recovery strategy mainly relies on higher knee and hip joints moments. However, as these changes are likely energy demanding, they would have been dampened by Slip10 if they were not necessary or did not provide some margin of safety in recovery. Therefore, although these increased internal joint moments were not correlated with improved balance as measured in this study (MoS or n_steps_), they might be correlated with other markers of balance. These adjustments may have been maintained because they had a positive impact on the vertical state of the COM rather than its horizontal (anterior posterior) state, as evaluated in this study (MoS), or on the regulation of the whole-body angular momentum. Therefore, the optimal recovery strategy described here only reflects the optimal strategy used to recover balance as measured by the anterior-posterior MoS, and other factors might affect dynamic stability.

Contrary to previous research quantifying balance recovery in static (Onambele et al., 2006) and dynamic conditions (Bierbaum et al., 2010; Pai et al., 2010; Konig et al., 2019b; McCrum et al., 2020) in YAs and OAs, we did not detect an effect of age on the balance ability of the participants (neither on MoS nor on n_steps_). One possibility for this lack of difference between the age groups could be that the perturbation triggered in this study did not present a mechanical demand high enough to discriminate the two groups. Indeed, although the MoS was lower than in normal condition it remained, on average, positive. Despite this lack of significant effect of age, trends were apparent on both the balance and the recovery strategy developed by the participants. We have previously shown that the MoS of YAs was lower than normal up to the fourth recovery step on the first exposure to a backward slip-like perturbation (Debelle et al., 2020); whereas here, the results show that when both the age groups are analysed together, participants need on average six recovery steps to return to normal MoS, indicating a tendency from OAs to be less stable than the YAs. Despite this tendency, the lack of significant age effect refutes the hypothesis that the number of recovery steps required to recover balance would be greater in OAs than in YAs. The OAs also tended to be grouped towards the low end of the correlation figures between kinetic or temporo-spatial variables and MoS (Figures 6B, 10B). Another explanation for the lack of differences between the OAs and the YAs in this study might simply be that the OAs we recruited were healthy, able to walk unassisted, and of relatively young age (62.4 ± 6.6 years), which may have shifted the results towards an undetectable effect of age. Therefore, caution should be exercised before extrapolating these results to frailer populations.

Regardless of age, participants' balance (MoS and n_steps_) improved with repeated exposures to backward slip-like perturbations. This is consistent with findings that ageing does not affect the capacity to learn new motor tasks per se (Bock and Schneider, 2002), and functionally that both YAs and OAs can improve their balance when exposed to repeated perturbations (Bierbaum et al., 2010; Pai et al., 2010; Konig et al., 2019b; McCrum et al., 2020). We observed high inter-individual variability in our results (Figure 3B), which could suggest a need for further training in participants performing poorly to achieve the same performance levels than the most proficient ones. Also, the MoS was measured as the distance between the anterior boundary of the BoS and the extrapolated centre of mass. This is based on the false assumption that the centre of pressure can travel infinitely fast (Hof and Curtze, 2016) and therefore overestimates the location of the anterior boundary of the BoS, which leads to an underestimation of the instability. An alternative way to measure the MoS would have been to downsize the BoS. To the best knowledge of the authors, there is no agreement yet on the proportion of the BoS that should be used to measure the MoS during perturbed walking conditions. However, results from the functional BoS measured during standing tasks show that the size of the functional BoS decreases with age (King et al., 1994; Tomita et al., 2021), which, if accounted for, could have affected the between-groups results. A further study is needed to fully understand which factors, if not age, can explain these limits in the improvement of balance with multiple exposures.

The time course by which participants reached the optimal recovery strategy, i.e., whether they gradually adjusted their response after each perturbation until reaching it or whether they selected and applied the optimal recovery strategy from pre-existing motor programs following repeated perturbations, was not investigated in this study. However, the results on balance (MoS and n_steps_) suggest an improvement with repeated slips within one session, which is consistent with previous studies on different kinds of gait perturbations (Pai et al., 2010; Konig et al., 2019b). Contradictory results show that the adaptation from repeated forward slips might only happen after an initial observational stage of three perturbations, in which the activity of the prefrontal cortex and the kinematics response to the perturbations were not modified (Lee et al., 2020). Therefore, further study is necessary to understand the time course of strategy adjustment, which is important to optimise the delivery of interventions utilising this approach.

Our results indicate a stabilisation of the effect no later than Slip06, but the number of repetitions required to provide a lasting effect is not known. In this study, we observed a plateau in the MoS improvement past the sixth slip, and from the third slip the number of steps required to return to normal balance did not differ from Slip10. These results suggest that three to six perturbations might be enough to trigger an online learning effect for backward slip-like perturbations, which is consistent with findings on forward slips (Pai et al., 2010) and trips (Epro et al., 2018a). However, the large standard deviation found in this study on both the balance and the mechanisms of balance recovery suggests that this threshold might be individual dependent.

Similar to the online learning effect, the long-term retention of the balance improvements, which is outside the scope of this study, is also conditioned by the number of perturbations triggered. Indeed, a single perturbation was not enough to trigger a long-term retention (Konig et al., 2019a), but a small number of trials (*n* = 8) successfully induced a lasting improvement in balance (Epro et al., 2018b; Konig et al., 2019b). Additionally, balance ability improvements may be retained at least over 1 month for backward slips in YAs (McCrum et al., 2018), and 1 year for forward slips in OAs (Pai et al., 2014). Although OAs are able to retain the balance improvements from the perturbation training, Konig et al. (2019b) have found that they lose the benefits of the first session quicker than YAs: exposed again to a lab-induced trip 14 weeks following the initial training, OAs' MoS was significantly lower than during the last perturbation of the first session when the YAs did not display this drop. Further work is needed to understand what the optimal perturbation dose (Karamanidis et al., 2020) is, i.e., the threshold above which additional perturbations would not improve the balance further and would trigger long-term retention of balance improvements.

Other considerations that were outside the scope of this study, such as transferability and generalisability of task-specific interventions, should also be investigated. Evidence exists for OAs that an inter-limb transfer of backward slip-like perturbations is possible (McCrum et al., 2020); however, transfer to other mechanical tasks is yet to be investigated. To the knowledge of the authors, generalisation of the benefits from treadmill induced backward slips to overground backward slips has not been investigated yet, but encouraging results on forward slips show that within session and long-term generalisation of the balance improvement following treadmill-induced slips is possible, although not as efficacious as overground-slip training (Liu et al., 2020).

Some limitations exist in this study that should be taken into account. First, we used a fixed walking speed in this study (1.2 m·s^−1^); therefore, because step length and stability (Bhatt et al., 2005) depend on walking speed, caution should be used when extrapolating the results to other walking speeds. However, as OAs have been shown to improve their balance recovery with repeated perturbations in self-selected (Bhatt et al., 2006), fixed (Epro et al., 2018a), and stability-normalised (McCrum et al., 2020) walking speeds, we are confident that the conclusions on balance improvements with repeated backward slip-like perturbations are not limited to this specific speed. Second, we did not find a significant correlation between the changes in kinetic (GRF angle and ankle moment) or temporo-spatial (step length) variables observed during the perturbed step (Pert) and the balance of the first recovery step (Rec1), which is probably even more important than Rec2 for fall avoidance. Therefore, other factors not investigated in the present study, such as participants' ankle plantarflexor and knee extensor muscles' strength and associated tendons' stiffness might be of significant importance in fall avoidance during the first recovery step. This lack of correlation between the mechanics of recovery during Pert and the balance of Rec1 might also be explained by the concomitance of the changes in these kinetic and temporo-spatial variables and the belt acceleration. Whether the observed changes (compared with Normal) are linked to an actual attempt to maintain a stable balance or to the belt acceleration (and therefore centre of pressure displacement) remains unknown. Third, as visual inspection of the moment-time curves did not identify notable changes in the timing of peak moments during the perturbed step or the first recovery step, we did not study the changes in the sequential organisation of the joint moments and how they may have affected the balance on the following steps. However, as the onset of knee moment generation seems to discriminate older fallers from young adults following trips (Pijnappels et al., 2005b), the timing of moment generation should be accounted for in future studies. Fourth, we took great care in recording baseline data during completely normal walking (no lateral or anterior-posterior displacements of the participants were visually observed) and used an average of five steps after a period of familiarisation, which might not be representative of the actual variability of the MoS during Normal condition. Using only five steps to measure the MoS in Normal conditions, we could have overestimated the MoS variability and therefore underestimated the number of participants who did not reach 1SD from Normal balance following the slips (n_steps_). However, participants' MoS variability ranged from 0.4 to 3.1 cm, which is not larger than the variability reported by McCrum et al. (2020), which ranged from ~1 to ~3.5 cm and was measured over 10 consecutive unperturbed steps. This, and recent observations by Fallahtafti et al. (2021) showing that treadmill walking leads to lower MoS variability compared with overground walking, suggest that the within-subjects MoS variability might not have been overestimated but rather underestimated, which may explain the large number of participants who did not return to stable gait by the 15th step in this study (*n* = 14 following the first slip). Careful considerations should be made concerning the number of steps used to determine the MoS variability in future studies, particularly when transferring from treadmill to overground tasks. Lastly, we found that participants did not make anticipatory adjustments in their MoS prior to Slip01, which is consistent with results reported on predictive changes in balance in unexpected perturbations (Okubo et al., 2018), did not anticipate the exact timing of the perturbation (no difference between Pre2 and Pre1 neither for Slip01 nor Slip10), but that following repeated exposures to backward slip-like perturbations, participants developed a more conservative gait pattern (increased MoS in Pre2 and Pre1 of Slip10), which is consistent with previous reports for trips (Wang et al., 2020) and forward slips (Pavol et al., 2004; Heiden et al., 2006; Lawrence et al., 2015). We found significant positive correlations between the MoS in Slip10_Pre2 and Slip10_Pre1 and the MoS of the first recovery step, which suggest, as already demonstrated by Bhatt et al. (2006), that the anticipatory adjustments in balance modulate the reactive ones, and possibly the outcome of the perturbation (fall or recovery). Therefore, the generalisability of our findings to recovery from real-world backward slips, for which there is likely no balance adjustment prior to the actual perturbation, might be dampened. This is a problem for any fall prevention intervention that utilises repeated perturbation exposures. However, the results reported by Pai et al. (2014) are encouraging, as OAs exposed to repeated (*n* = 24) lab-induced forward slips were found to be 2.3 times less likely to fall within a year than those exposed to a single slip.

To summarise, we showed that independent of age, participants improved their balance with repeated exposure to backward slip-like perturbations. We found that the length of the first recovery step following the slip is an important variable for the improvement of balance recovery and was optimised with repeated slips by returning it close to normal levels. As this variable can easily be measured and controlled, instructing OAs to increase their step length when their gait is perturbed may help them recover their balance and potentially avoid falling more effectively.

## Data Availability Statement

The raw data supporting the conclusions of this article will be made available by the authors, without undue reservation.

## Ethics Statement

The studies involving human participants were reviewed and approved by Liverpool John Moores University ethics committee National Health Service (NHS) ethics committee (18/NW/0700). The patients/participants provided their written informed consent to participate in this study.

## Author Contributions

HD, TO'B, and CM: contributed to the conception and design of the research, interpretation of the results, edited and revised the manuscript, and agreed to manuscript submission for publication. HD: data acquisition and data analysis and drafted the manuscript. All authors contributed to the article and approved the submitted version.

## Funding

This study was co-funded by Minerva Research Labs Ltd. (London, United Kingdom) and Liverpool John Moores University (Liverpool, United Kingdom). The funders were not involved in the study design, collection, analysis, interpretation of data, the writing of this article or the decision to submit it for publication.

## Conflict of Interest

The authors declare that the research was conducted in the absence of any commercial or financial relationships that could be construed as a potential conflict of interest.

## Publisher's Note

All claims expressed in this article are solely those of the authors and do not necessarily represent those of their affiliated organizations, or those of the publisher, the editors and the reviewers. Any product that may be evaluated in this article, or claim that may be made by its manufacturer, is not guaranteed or endorsed by the publisher.
