# Supplementary material for: Biomechanical Mechanisms of Improved Balance Recovery to Repeated Backward Slips Simulated by Treadmill Belt Accelerations in Young and Older Adults
Source: Front Sports Act Living. 2021 Sep 21;3:708929. doi: 10.3389/fspor.2021.708929 (PMC8490723; doi:10.3389/fspor.2021.708929)
Supplement: Supplementary file 1 [file Data_Sheet_1.docx]

Supplementary Material

# Young and older adults’ data from main manuscript’s figures


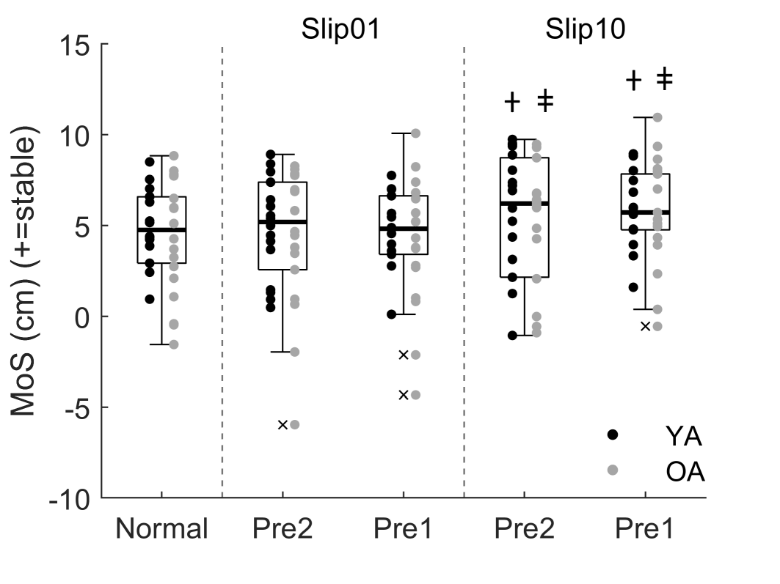
To improve the main manuscript’s readability, most figures were plotted as the average of both groups. Below are the same figures plotted as the average of each group where appropriate, or with participants’ data points added.

Supplementary figure 1: *Main manuscript’s figure 2.A.* Boxplots of the margin of stability (MoS) for all participants in the two steps prior to Slip01 and Slip10 (Pre2 and Pre1). Thick horizontal black lines: median, thin horizontal black line: first and third quartiles, ×: outliers. Ⴕ: Slip10 significantly higher than Normal (p≤0.005), ǂ: Slip10 significantly higher than Slip01 (p≤0.023). Black circles: young adults, grey circles: older adults. No significant effect of Age existed.


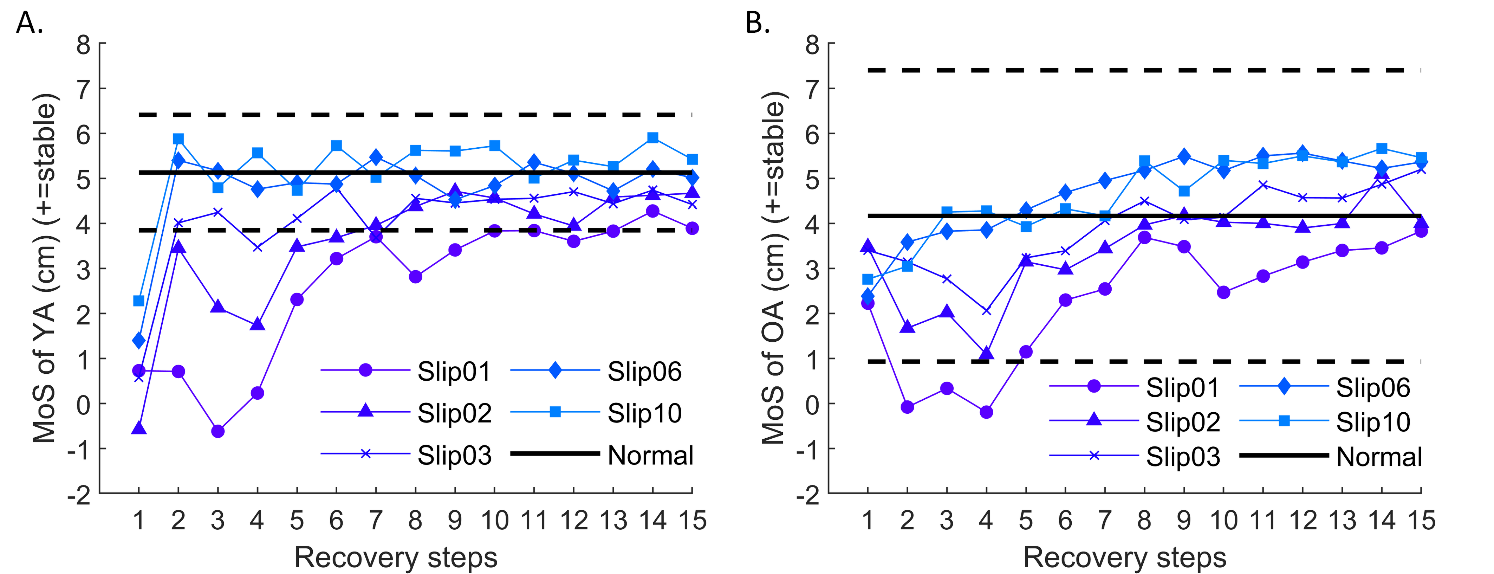

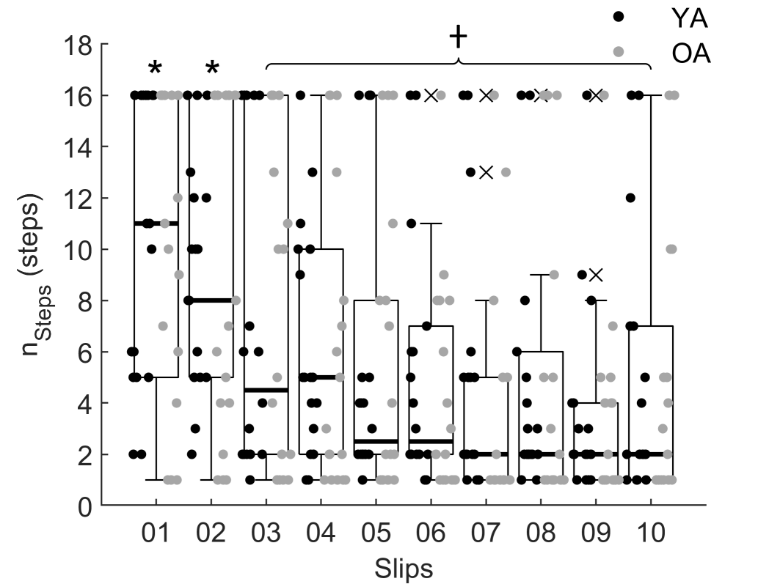


Supplementary figure 3: *Main manuscript’s figure 3.B.* Boxplots of n_steps_ (i.e. first step of at least 3 consecutive steps back to ± 1SD of Normal MoS) from Slip01 and Slip10. Thick horizontal black lines: median, thin horizontal black line: first and third quartiles, ×: outliers. *: significantly larger than Slip10, p≤0.0011, Ⴕ: significantly lower than Slip01, p≤0.0011. Black data points: young adults, grey data points: older adults. No significant effect of Age existed.

Supplementary figure 2: *Main manuscript’s figure 3.A.* (A) Average margin of stability for YA for the fifteen recovery steps recorded for Slip01, Slip02, Slip03, Slip06 and Slip10. Solid and dotted horizontal black lines represent Normal ± 1SD, respectively. (B) Average margin of stability for OA for the fifteen recovery steps recorded for Slip01, Slip02, Slip03, Slip06 and Slip10. Solid and dotted horizontal black lines: Normal ± 1SD for the age group, respectively. No significant effect of Age existed.


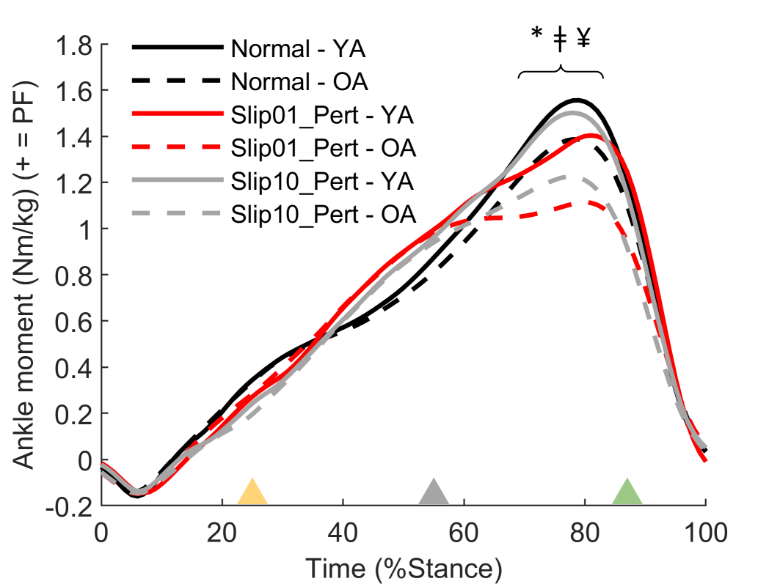

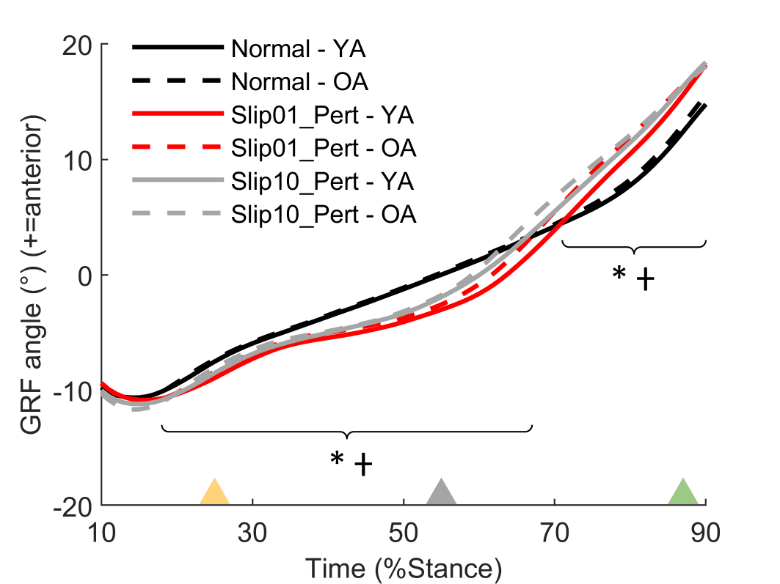


Supplementary figure 5: *Main manuscript’s figures 5 and 6.A.* Average ankle moment, effect of condition between Normal (black lines), Slip01_Pert (red lines), and Slip10_Pert (grey lines), for YA (solid lines) and OA (dotted lines). *: significant difference between Normal and Slip01 (69 to 83% stance, p=0.002), ǂ: significant difference between Slip01 and Slip10 (70 to 79% stance, p<0.001), ¥: significant difference between YA and OA for Slip10_Pert (69 to 79% stance, p=0.004). Yellow triangle: beginning of belt acceleration, grey triangle: peak belt speed, green triangle: belt speed returns to 1.2 m·s^-1^.

Supplementary figure 4: *Main manuscript’s figure 4.A.* Average GRF_θ_ (°), effect of condition between Normal (black lines), Slip01_Pert (red lines) and Slip10_Pert (grey lines), for YA (solid lines) and OA (dotted lines). *: Slip01 significantly different from Normal p<0.001 from 18 to 67% and from 71 to 90% of stance, Ⴕ: Slip10 significantly different from Normal p<0.001 from 15 to 60% and from 68 to 90% of stance. Yellow triangle: beginning of belt acceleration, grey triangle: peak belt speed, green triangle: belt speed returns to 1.2 m·s^-1^. No significant effect of Age existed.


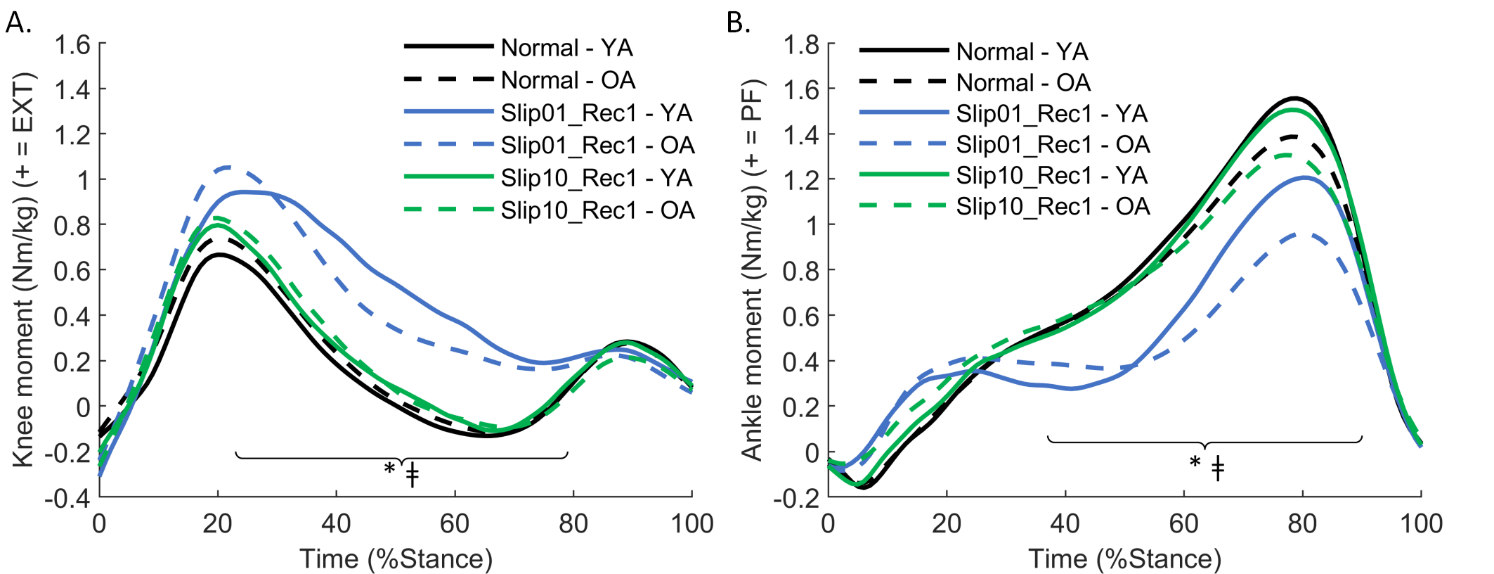

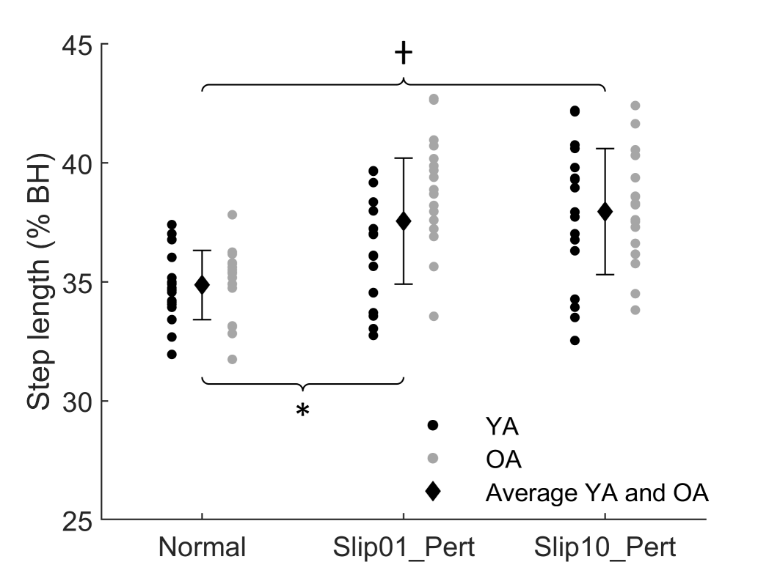


Supplementary figure 7: *Main manuscript’s figure 8.A and 8.B.* Average knee moment: effect of condition between Normal (black lines), Slip01_Rec1 (blue lines), and Slip10_Rec1 (green lines) for YA (solid lines) and OA (dotted lines). *: significant difference between Normal and Slip01 (23 to 79% stance p=0.002), ǂ: significant difference between Slip01 and Slip10 (32 to 80% stance p=0.002). (B) Average ankle moment: effect of condition between Normal(black lines), Slip01_Rec1 (blue lines), and Slip10_Rec1 (green lines) for YA (solid lines) and OA (dotted lines). *, significant difference between Normal and Slip01 (37 to 90% stance p=0.002), ǂ: significant difference between Slip01 and Slip10 (34 to 89% stance p<0.001). No significant effect of Age existed.

Supplementary figure 6: *Main manuscript’s figure 7.A.* Step length of Slip01_Pert and Slip10_Pert compared to Normal (average ± SD as error bars), *: significant difference between Slip01 and Normal, p<0.001. Ⴕ: significant difference between Slip10 and Normal, p<0.001. Diamond shape markers: average of both YA and OA, black circles: YA data points, grey circles: OA data points. No significant effect of Age existed.

#
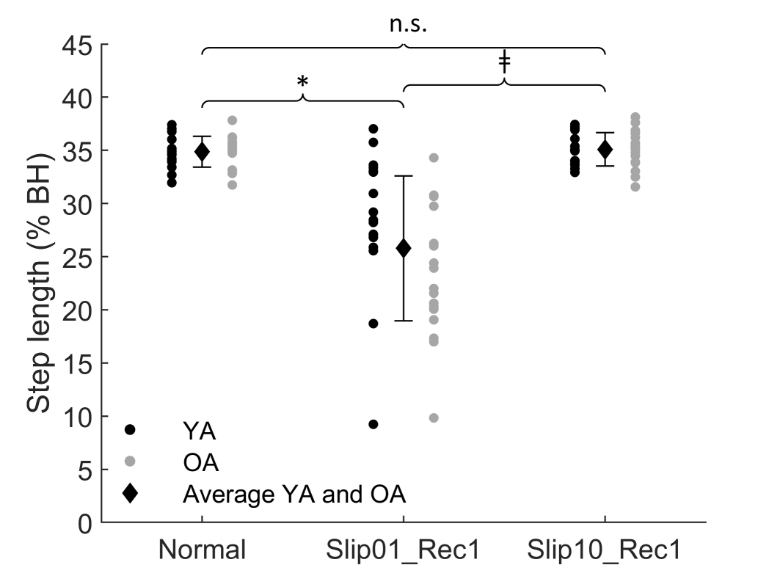


Supplementary figure 8: *Main manuscript’s figure 10.A.* Step length of Slip01_Rec1 and Slip10_Rec1 compared to Normal (average ± SD as error bars). *: significantly shorter than Normal, p<0.001, ǂ: Slip10 significantly longer than Slip01 p<0.001, n.s.: no significant difference between conditions. Diamond shape markers: average of both YA and OA, black circles: YA data points, grey circles: OA data points. No significant effect of Age existed.

#
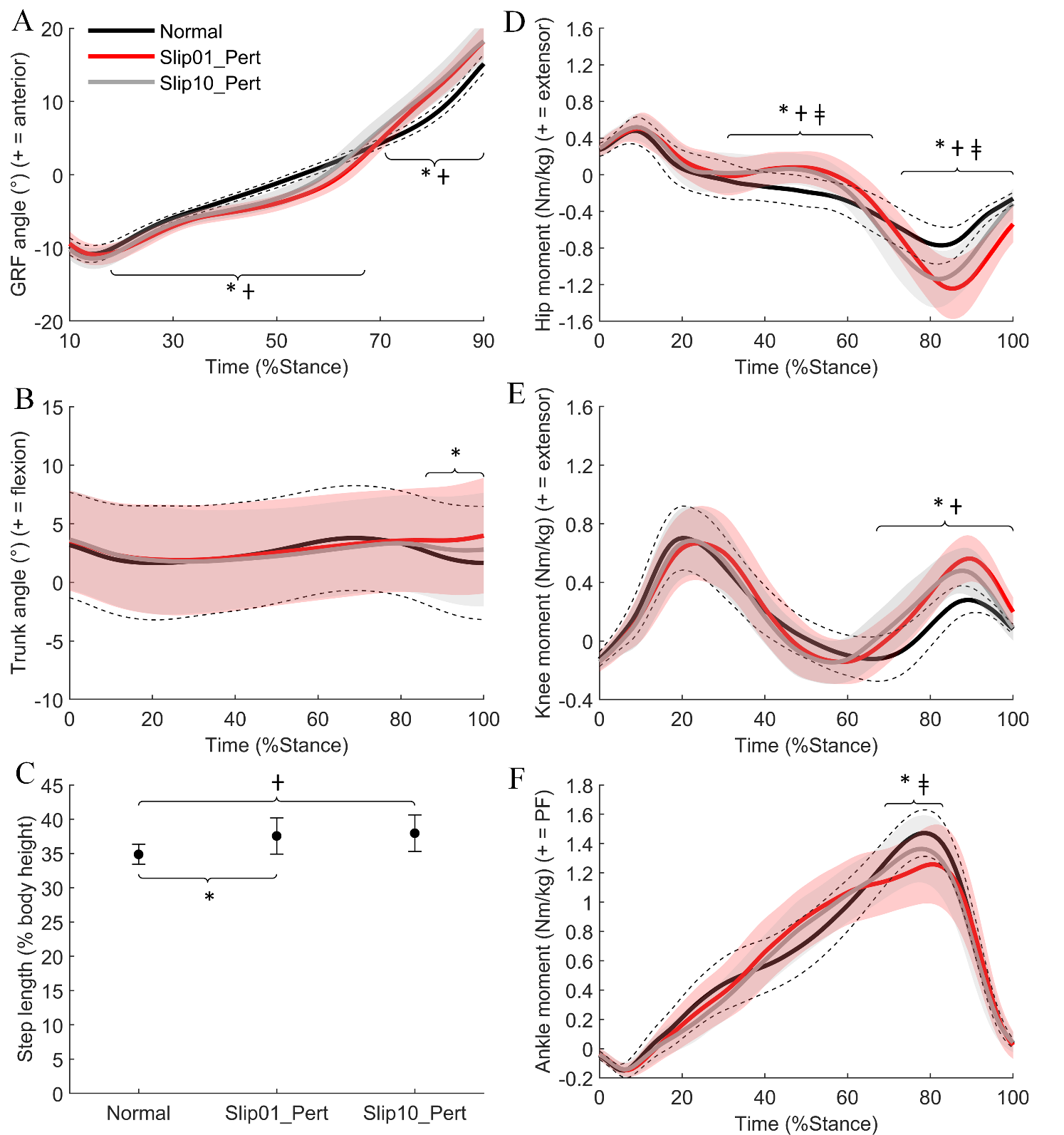
Kinetics and kinematics of perturbed step

Supplementary figure 9: (A) GRF angle in Normal, Slip01_Pert and Slip10_Pert, (B) Trunk angle, (C) Step length, (D) Hip moments, (E) Knee moments, (F) Ankle moments. * Significant difference between Normal and Slip01, Ⴕ Significant difference between Normal and Slip10, ǂ Significant difference between Slip01 and Slip10.

Supplementary table 1: Timing of significant difference between conditions, in % of stance, for the perturbed step: * significant difference between Normal and Slip01, Ⴕ significant difference between Normal and Slip10, ǂ significant difference between Slip01 and Slip10.

|  |  | * Normal and Slip01 | Ⴕ Normal and Slip10 | ǂ Slip01 and Slip10 |
| --- | --- | --- | --- | --- |
| Perturbed step | Hip Moment | 31 to 66 | 29 to 61 | 57 to 75 |
|  |  | 73 to 100 | 67 to 98 | 90 to 100 |
|  | Knee moment | 67 to 100 | 65 to 95 | n.s. |
|  | Ankle moment | 69 to 83 | n.s. | 70 to 79 |
|  | GRF angle | 18 to 67 | 15 to 60 | n.s. |
|  |  | 71 to 90 | 68 to 90 | n.s. |
|  | Trunk angle | 86 to 100 | n.s. | n.s. |

# Kinetics and kinematics of first recovery step


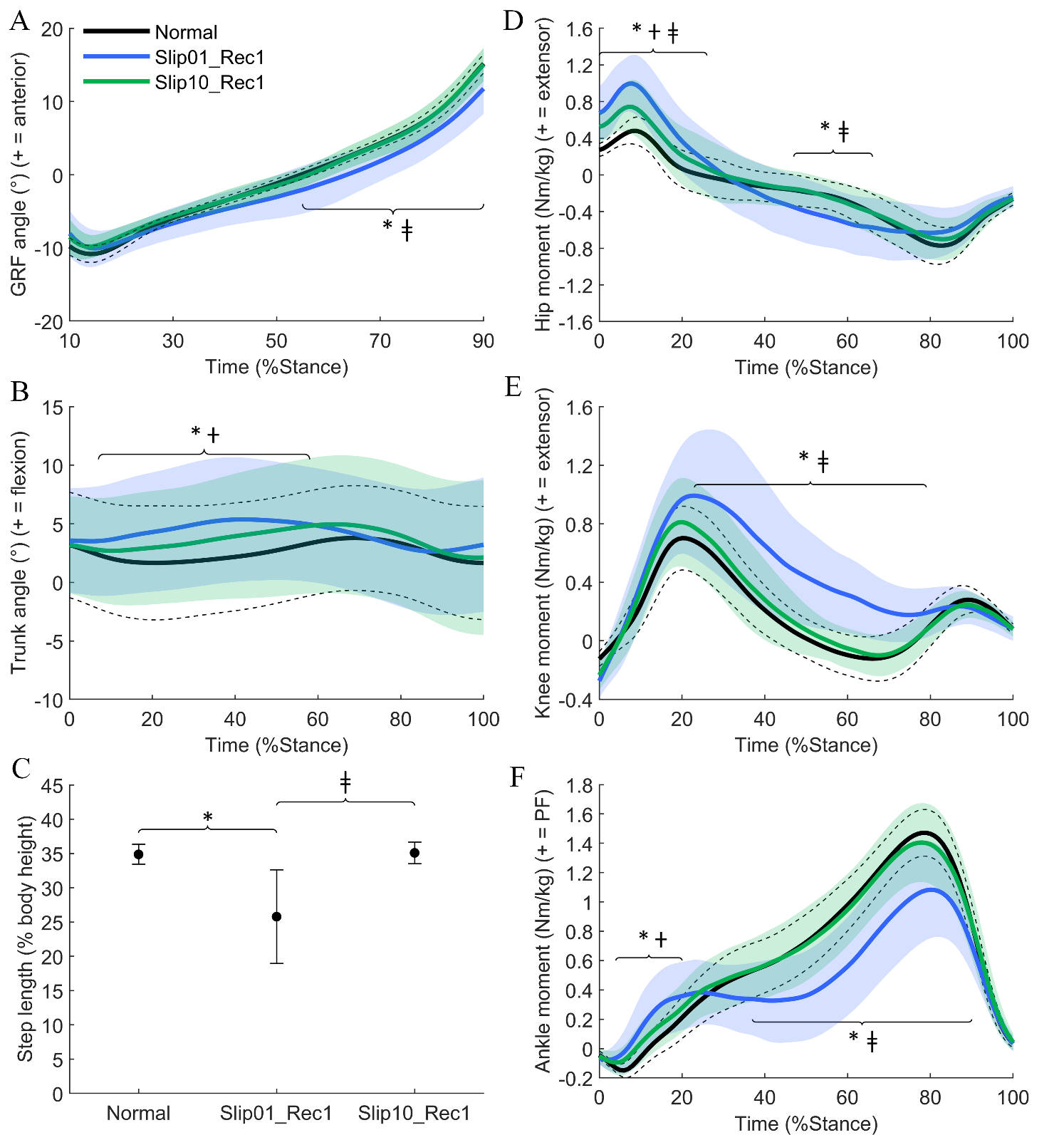


Supplementary figure 10: (A) GRF angle in Normal, Slip01_Rec1 and Slip10_ Rec1, (B) Trunk angle, (C) Step length, (D) Hip moments, (E) Knee moments, (F) Ankle moments. * Significant difference between Normal and Slip01, Ⴕ Significant difference between Normal and Slip10, ǂ Significant difference between Slip01 and Slip10.

Supplementary table 2: Timing of significant difference between conditions, in % of stance, for the first recovery step: * significant difference between Normal and Slip01, Ⴕ significant difference between Normal and Slip10, ǂ significant difference between Slip01 and Slip10.

|  |  | * Normal and Slip01 | Ⴕ Normal and Slip10 | ǂ Slip01 and Slip10 |
| --- | --- | --- | --- | --- |
| First recovery step | Hip Moment | 0 to 26 | 0 to 22 | 5 to 17 |
|  |  | 47 to 66 | n.s. | 46 to 65 |
|  | Knee moment | 23 to 79 | n.s. | 32 to 80 |
|  | Ankle moment | 4 to 20 | 5 to 13 | n.s. |
|  |  | 37 to 90 | n.s. | 34 to 89 |
|  | GRF angle | 55 to 90 | n.s. | 62 to 90 |
|  | Trunk angle | 7 to 58 | 20 to 42 | n.s. |

#
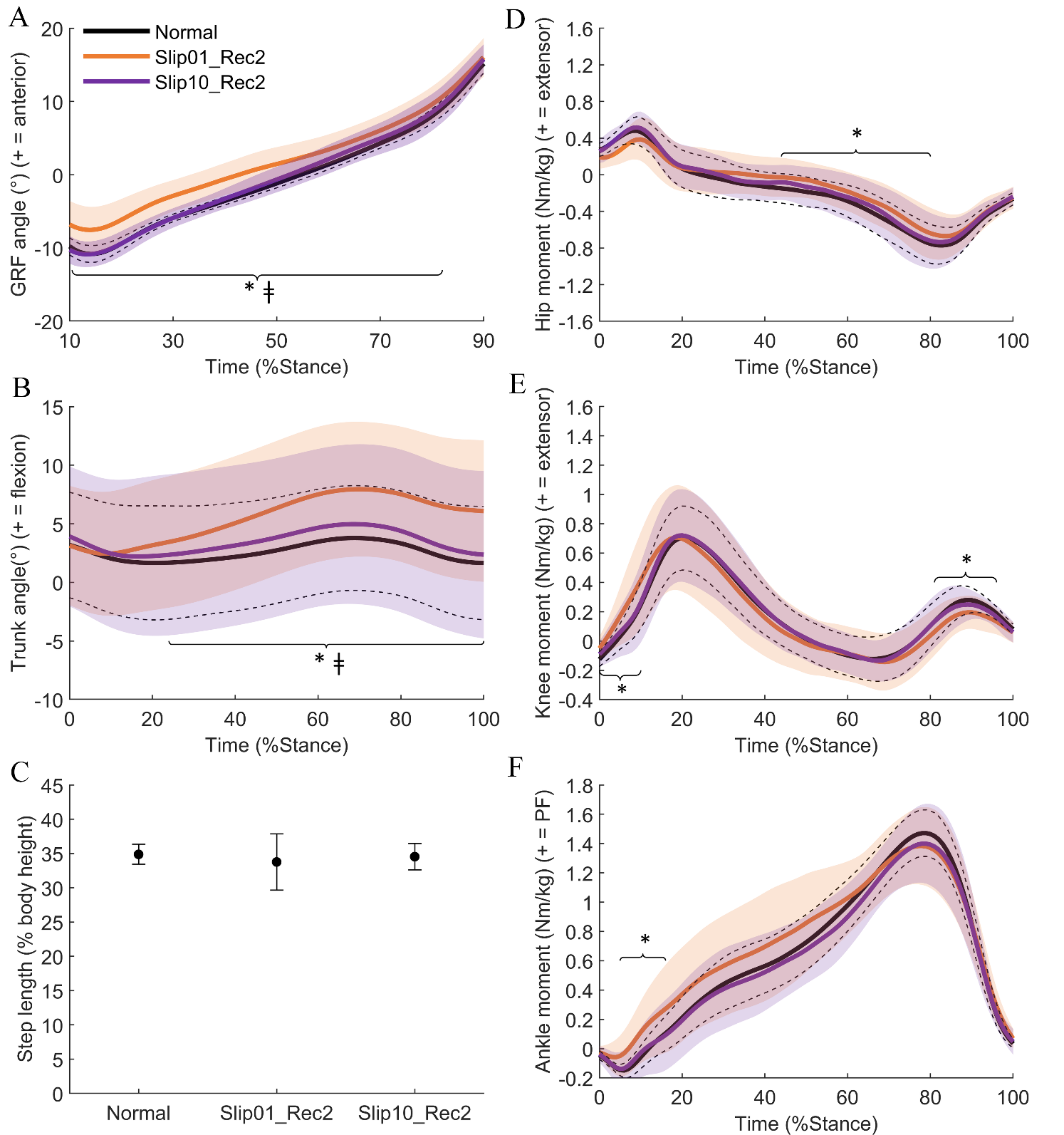
Kinetics and kinematics of second recovery step

Supplementary figure 11: (A) GRF angle in Normal, Slip01_Rec2 and Slip10_ Rec2, (B) Trunk angle, (C) Step length, (D) Hip moments, (E) Knee moments, (F) Ankle moments. * Significant difference between Normal and Slip01, Ⴕ Significant difference between Normal and Slip10, ǂ Significant difference between Slip01 and Slip10.

Supplementary table 3: Timing of significant difference between conditions, in % of stance, for the second recovery step: * significant difference between Normal and Slip01, Ⴕ significant difference between Normal and Slip10, ǂ significant difference between Slip01 and Slip10.

|  |  | * Normal and Slip01 | Ⴕ Normal and Slip10 | ǂ Slip01 and Slip10 |
| --- | --- | --- | --- | --- |
| Second recovery step | Hip moment | 44 to 80 | n.s. | n.s. |
|  | Knee moment | 0 to 10 | n.s. | n.s. |
|  |  | 81 to 96 | n.s. | n.s. |
|  | Ankle moment | 5 to 16 | n.s. | n.s. |
|  | GRF angle | 10 to 82 | n.s. | 10 to 56 |
|  | Trunk angle | 24 to 100 | n.s. | 42 to 100 |
